# An Operationally Simple Approach to Indole Derivatives from 2-Alkenylanilines Utilizing an Oxidation–Intramolecular Cyclization–Elimination Sequence

**DOI:** 10.3390/molecules28247968

**Published:** 2023-12-06

**Authors:** Lauren N. Hines, Jacob R. King, Alex C. Atwood, Rachel M. Chapman, Matthew B. Griffey, Christine R. Tutwiler, Christopher Jon Monceaux

**Affiliations:** Department of Chemistry, Radford University, Radford, VA 24142, USAaatwood@radford.edu (A.C.A.);

**Keywords:** metal-free indole synthesis, epoxidation, intramolecular cyclization

## Abstract

Herein we describe a novel route to indole derivatives from a variety of *N*-substituted 2-alkenylanilines. This route features three operationally simple steps: (1) oxidation to convert *N*-substituted 2-alkenylanilines into epoxide intermediates, (2) intramolecular cyclization, and (3) the acid-catalyzed elimination of water.

## 1. Introduction

**Scheme 1:**
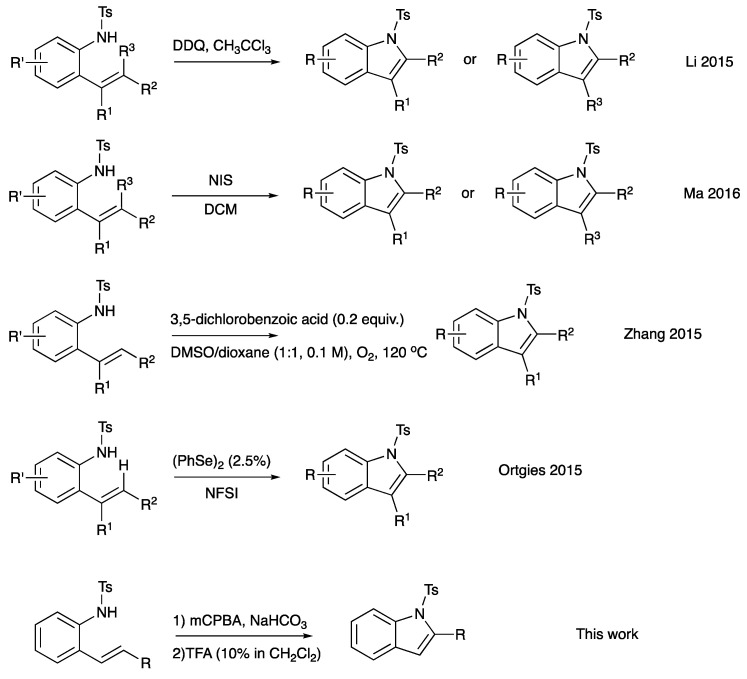
Selected examples of indole synthesis from 2-alkenylanilines [8,9,10,11].

The indole motif is accepted as a privileged scaffold; indoles are capable of acting as ligands for a variety of biological targets [1]. Indoles are found in a wide range of natural sources, including plants, animals, and microorganisms. Many indole-containing compounds exhibit significant biological activity [1]. For example, several alkaloids containing the indole ring, such as serotonin, tryptamine, and ergotamine, play essential roles in the regulation of physiological processes and can have profound effects on human health and behavior. Indoles are also found in a variety of pharmaceuticals, including antipsychotic, antidepressant, and antimicrobial drugs. In addition to their biological importance, indoles serve as versatile building blocks in organic synthesis. The indole ring can be functionalized and modified to create a wide range of chemical compounds with diverse properties. Unsurprisingly, there is no shortage of methods to synthesize indole derivatives, and yet there still remains great interest in new and streamlined methods of synthesizing indole derivatives. The conversion of 2-alkenylanilines into indoles has emerged as a straightforward route, owing to the extensive availability of both anilines and alkenes (or styrenes). One such route is C-H amination through transition metal catalysts [2,3,4,5,6]. Recently, methods avoiding the use of metals in cyclization have attracted much attention. A few notable examples are Scheme 1, the use of DDQ mediation, NIS mediation, selenium-catalyzed NFSI oxidation, and 3,5-dichlorobenozic acid catalysis with molecular oxygen as the oxidant [7,8,9,10,11,12,13]. Herein, we report an additional method to the metal-free C-H aminations listed above. Our 2-alkenylaniline undergoes epoxidation with *m*-CPBA to form a metastable epoxide intermediate that then undergoes acid-catalyzed cyclization–dehydration to furnish a C1-substituted indole derivative (Scheme 1**,** final entry).

## 2. Discussion

**Scheme 2:**
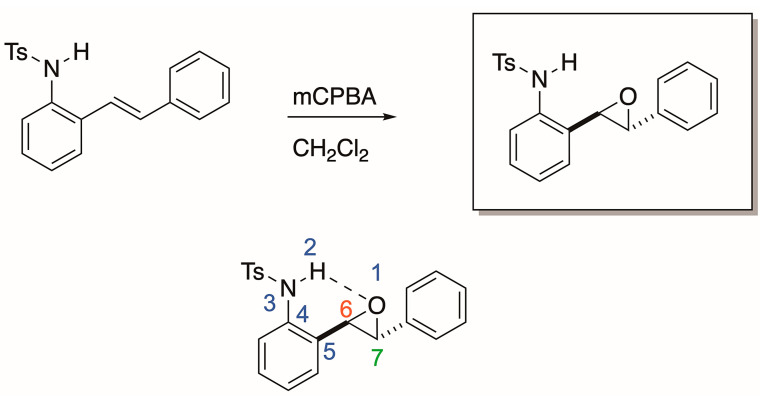
Proposed regioselective nucleophilic opening of our 2-alkenylaniline via intramolecular N-H Lewis acid activation.

**Scheme 3:**
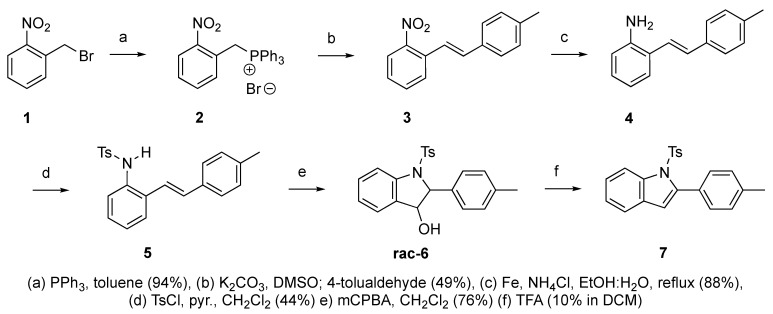
Synthetic route to indole derivative **7** from 2-alkenylaniline **4**.

Our group was initially interested in the synthesis of epoxides derived from 2-alkenylanilines (Scheme 2). We proposed that N-H could activate the epoxide for regioselective nucleophilic opening, which is an area of established and ongoing research in our laboratory [14]. We propose that an intramolecular Lewis acid-catalyzed opening of the epoxide will only be viable if a nucleophilic attack occurs at carbon 7 (labeled in red, Scheme 2). In our attempt to synthesize the epoxide, we treated the 2-alkenylaniline (Scheme 2) with *m*CPBA in dichloromethane. To our surprise, upon analysis with ^1^H NMR, we had, in fact, formed the indolin-3-ol compound (**6**, Scheme 3). We supposed that this compound could eliminate water quite easily to form an indole. Indeed, upon heating the tube containing the sample in the NMR to 40 °C for 12 h, indolin-3-ol eliminated water to form the indole (**7**, Scheme 3). While not our original intent, we set out to expand this chemistry to establish a new and operationally simple pathway to indoles from 2-alkenylanilines.

**Scheme 4:**
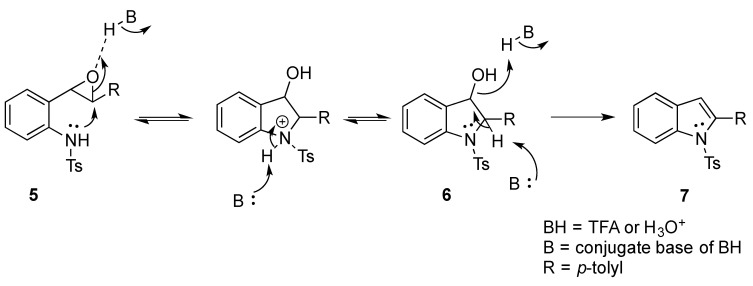
Proposed mechanism for the epoxidation–cyclization–elimination cascade.

We set out to arrive at these epoxides via the epoxidation of (*E*)-*N*-Ts-styrylaniline (**5**, Scheme 3). Because our initial interest was the regioselective opening of epoxides, we desired to have both *E*- and *Z*-diastereomers. Hence, our initial synthetic route began with forming phosphonium salt (**2**) from commercially available 2-nitrobenzyl bromide (**1**). Using 4-tolualdehyde in a Wittig reaction furnished both *E*- and *Z*-diastereomers, and we were able to isolate the *E*-diastereomer (**3**) utilizing silica gel flash chromatography. An Fe-catalyzed reduction furnished styrylaniline (**4**) in good yield, which was followed by forming sulfonamide (**5**). At this point, we were prepared to perform the epoxidation. We chose to utilize the well-known method of stirring *m*CPBA in dichloromethane for a period of 18 h. TLC indicated complete consumption of the alkene (**5**) while also providing a clean conversion into a more polar product. However, upon ^1^H NMR analysis, we could not find the proton resonance corresponding to the sulfonamide (N-H). Additionally, the chemical shifts in resonances for the supposed oxirane protons and carbons were difficult to rationalize. We then proposed that the epoxide was undergoing an intramolecular cyclization into an indolin-3-ol structure (**6**), which was consistent with our NMR data. We also presumed that since elimination would aromatize the nitrogen-containing ring, the elimination reaction should occur with mild heating. Indeed, heating the sample to 40 °C while still in the NMR tube (using the NMR spectrometer (Bruker, Billerica, MA, USA) as an oven), we observed the formation of an indole (**7**) that was nearly complete by 12 h at 40 °C. Additionally, we presumed that an acid could also catalyze the elimination of **6** to **7**. We prepared a solution of **6** in dichloromethane (0.05 M) and added TFA (10% by volume). Instantaneously, the color of the solution changed, and TLC indicated a complete conversion into the indole (**7**). We propose a mechanism for this acid-catalyzed intramolecular cyclization–elimination sequence (Scheme 4).

**Scheme 5:**
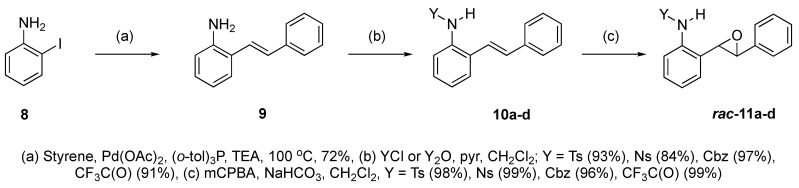
Synthesis of 2-alkenylaniline epoxides.

At the time of this finding, we changed our synthetic route to *N*-substituted styrylanilines to one that was much shorter and more efficient (Scheme 5). Heck coupling between 2-iodoaniline (**8**, Scheme 5) and styrene provided *E*-2-styrylaniline (**9a**, Scheme 5) in one step without a detectable amount of *Z*-diastereomer. The nitrogen was then functionalized with a Ts (**10a**), Ns (**10b**), Cbz (**10c**), or trifluoroacetyl (**10d**) group. Cognizant that acidic conditions would catalyze the oxidation–cyclization–elimination sequence observed in **5** → **7**, we added an excess of NaHCO_3_ to the epoxidation reaction. We were pleased that we were able to isolate relatively pure epoxide intermediates (**11a**–**d**) in very high yields without any purification (Scheme 5).

**Scheme 6:**
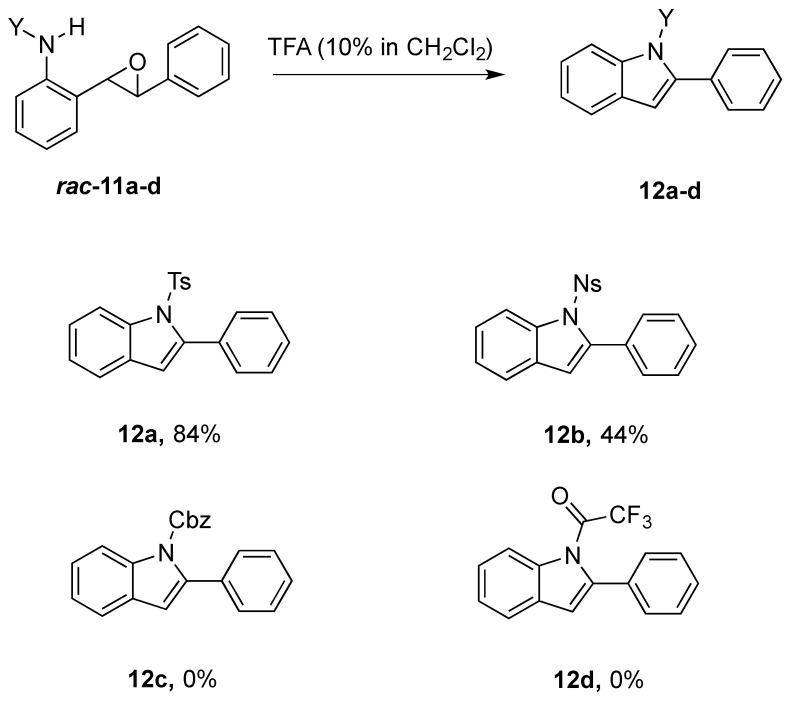
Scope of cyclization-elimination reaction with varying *N*-protecting groups.

The crude epoxides were then dissolved in dichloromethane and treated with TFA (Scheme 6). Compound **11a** (Y = Ts) reacted and provided indole **12a** very quickly. However, **11b** (Y = Ns) reacted much slower and, in fact, was not complete after 48 h. This is consistent with our proposed mechanism, as the Ns group, being much more electron-withdrawing, reduces the nucleophilicity of the nitrogen atom. Unfortunately, compounds **12c** and **12d** provided complex mixtures. We suspect that a competing intramolecular cyclization reaction through the carbamate/amide oxygen could be operating, and this is a current area of further investigation in our research.

After our survey of these *N*-protecting groups, we decided that the Ts group provided the best properties for our route to indoles from 2-alkenylanilines.

**Scheme 7:**
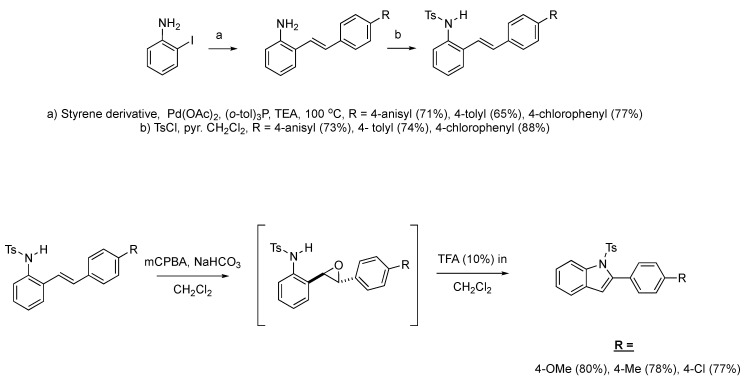
Additional 2-alkenylanilines used in the formation of C1-substituted indole derivatives.

We then set out to extend this chemistry to investigate its scope by using different styrenes in the Heck coupling step. The three additional styrenes selected were 4-methyoxystyrene, 4-methylstyrene, and 4-chlorostyrene. The synthesis of these three indole derivatives is summarized in Scheme 7.

## 3. Materials and Methods

General. Starting materials were purchased from commercial suppliers and used as received unless otherwise noted. Solvents were used as received from Fisher Scientific. TLC was performed using 200 μm Silica XG Plates with UV254 from Sorbent Technologies. Column chromatography was performed using 60A, 40–63 μm silica gel from Sorbent Technologies. ^1^H, ^13^C, and ^31^F NMR spectra were recorded at 400, 101, and 376 MHz, respectively, on a Bruker Avance III HD instrument (see Supplementary Materials). Chemical shifts are reported in ppm using the known chemical shift of the indicated solvent as an internal reference. The following abbreviations are used to indicate coupling: s (singlet), d (doublet), t (triplet), q (quartet), qt (quintet), and br. (broad).

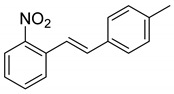

  *(E)-1-(4-methylstyryl)-2-nitrobenzene* (**3**): A procedure created by Nomura et al. was adapted for our synthesis [15]. To a 50 mL round-bottom flask containing (2-nitrobenzyl)triphenylphosphonium bromide (1.89 g, 3.94 mmol), we added DMSO (12 mL) and K_2_CO_3_ (7.88 mmol, 1.09 g). This mixture was allowed to stir for 2 h. 4-Tolualdehyde (3.94 mmol, 464 uL) was then added to the stirred mixture via syringe. This mixture was allowed to stir for 18 h. TLC (10% Et_2_O in Hex) indicated complete consumption of the aldehyde. Two spots appeared above the baseline, which are likely the *E*- and *Z*-isomers of the product. The purple mixture was poured into water (15 mL) and then extracted with ethyl acetate (3 × 20 mL) and then Et_2_O (2 × 15 mL). The combined organic layers were dried over MgSO_4_, filtered, and concentrated. The residue was purified via column chromatography (5.5% EtOAc in Hex). A set of fractions containing more polar *E*-isomers was isolated and concentrated to provide a yellow oil (460 mg, 1.92 mmol, 48.7%). ^1^H NMR(CDCl_3_, 400 MHz): δ (ppm) 7.93 (dd, *J* = 8.2, 1.3 Hz, 1H), 7.75 (dd, *J* = 7.8, 1.3 Hz, 1H), 7.62–7.56 (m, 2H), 7.53 (d, *J* = 16.3 Hz, 2H), 7.42 (d, *J* = 8.1 Hz, 2H), 7.40–7.34 (m, 2H), 7.17 (d, *J* = 7.9 Hz, 2H), 7.05 (d, *J* = 16.1 Hz, 1H), 2.36 (s, 3H). ^13^C NMR(CDCl_3_, 101 MHz): δ (ppm) 148.18, 138.92, 134.07, 133.95, 133.39, 133.19, 129.72, 128.23, 127.91, 127.23, 124.95, 122.59, 21.54.

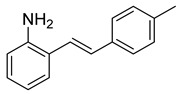

  *(E)-2-(4-methylstyryl)aniline* (**4**). A 3-neck 50 mL round-bottom flask was charged with the nitrobenzene derivative (**3**, 220 mg, 0.92 mmol) and absolute ethanol (6.7 mL). The mixture was heated slightly to dissolve the nitrobenzene derivative. Iron powder (154 mg, 2.76 mmol) and ammonium chloride (50 mg, 0.92 mmol) were then added followed by water (1.8 mL). (A white precipitate formed upon the addition of water that gave way to a solution near the point of reflux.) The system was fitted with a reflux condenser and was then purged with nitrogen for 5 min. The mixture was then heated to reflux by a heating mantle for 1.5 h. TLC indicated the complete consumption of the starting material. After cooling, 15 mL of EtOH was added to the mixture and was heated slightly to dissolve the organic materials. The suspension was filtered through a pad of celite, and the filtrate was concentrated to provide a yellow solid. The solid was dissolved in 15 mL of EtOAc and washed with 10 mL of sat’d NaHCO_3_. The aqueous layer was further extracted with EtOAc (2 × 15 mL). The combined organic layers were dried over MgSO_4_, filtered, and concentrated to provide a dark yellow solid (169.5 mg, 0.81 mmol, 88%) that was used without further purification. ^1^H NMR(CDCl_3_, 400 MHz): δ (ppm) 7.45–7.34 (m, 3H), 7.17–7.05 (m, 4H), 6.95 (d, *J* = 16.0 Hz, 1H), 6.84 (td, *J* = 7.5, 1.1 Hz, 1H), 6.77 (dd, *J* = 7.9, 1.2 Hz, 1H), 4.35 (br. s, 1H), 2.34 (s, 3H). ^13^C NMR(CDCl_3_, 101 MHz): δ (ppm) 142.69, 137.73, 134.95, 130.85, 129.60, 128.66, 127.36, 126.62, 125.04, 123.18, 120.23, 117.11, 21.47.

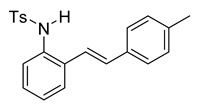

  To a solution of **4** (611 mg, 2.91 mmol) and pyridine (0.70 mL, 8.76 mmol, 3.0 equiv) in DCM (12 mL, 0.25 M), we added *p*-toluenesulfonyl chloride (668 mg, 3.5 mmol, 1.2 equiv.) at 0 °C. The reaction was stirred in an ice bath while warming to room temperature for 12 h. The reaction mixture was quenched by the addition of a saturated aqueous solution of NH_4_Cl, and then, the product was extracted with DCM (3 × 10 mL). The combined organic phase was washed with brine, dried over Na_2_SO_4_, filtered, and concentrated under reduced pressure. The residue was purified via flash column chromatography on silica gel (20% EtOAc in hexanes) to afford a white solid (788 mg, 2.17 mmol, 74%). ^1^H NMR(CDCl_3_, 400 MHz): δ (ppm) 7.54 (d, *J* = 8.3 Hz, 2H), 7.35 (ddd, *J* = 32.3, 7.0, 2.6 Hz, 2H), 7.18–7.03 (m, 8H), 6.72–6.58 (m, 2H), 6.36 (s, 1H), 2.30 (s, 3H), 2.24 (s, 3H). ^13^C NMR(CDCl3, 101 MHz): δ (ppm) 143.92, 138.18, 136.60, 133.88, 133.20, 133.14, 132.40, 129.67, 129.37, 128.26, 127.16, 126.98, 126.61, 126.56, 126.50, 121.49, 21.50, 21.30.

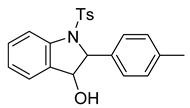

  *2-(p-tolyl)-1-tosylindolin-3-ol* (**6**). To a solution of the alkene (122 mg, 0.34 mmol) in dichloromethane (7 mL) under a stream of nitrogen, we added *m*-CPBA (70%, 165 mg, 0.67 mmol). The suspension was stirred at room temperature for 16 h. In the morning, the contents were poured into a flask containing 3 mL of NaHSO_3_ and 6 mL of NaHCO_3_. The biphasic solution was stirred vigorously for 10 min before pouring into a separatory funnel. The aqueous layer was extracted with dichloromethane (2 × 15 mL) and dried over Na_2_SO4. The solution was then filtered and concentrated to provide a solid residue. The residue was purified via silica gel flash chromatography (30% EtOAc in hexanes) to provide a white solid (96 mg, 0.25 mmol, 76%). ^1^H NMR(CDCl_3_, 400 MHz): δ (ppm) 7.75 (d, *J* = 8.2 Hz, 1H), 7.59–7.53 (m, 2H), 7.38–7.32 (m, 1H), 7.24–7.19 (m, 1H), 7.16–6.98 (m, 7H), 5.02 (d, *J* = 1.1 Hz, 1H), 4.67 (d, *J* = 1.2 Hz, 1H), 2.28 (s, 3H), 2.23 (s, 3H). ^13^C NMR(CDCl3, 101 MHz): δ (ppm) 144.25, 142.42, 137.67, 136.15, 134.63, 132.00, 130.84, 129.63, 129.41, 127.30, 126.23, 125.87, 124.93, 116.62, 79.05, 74.23, 21.56, 21.09.

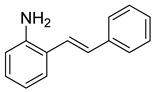

  *(E)-2-styrylaniline* (**9**). To a solution of 2-iodoaniline (1.04 g, 4.8 mmol, 1.0 eq.) in triethylamine (4.8 mL, 1.0 M), Pd(OAc)_2_ (11 mg, 0.047 mmol, 1 mol%), P(*o*-Tol)_3_ (115 mg, 0.38 mmol, 8 mol%), and styrene (593 mg, 5.7 mmol, 1.2 eq.) were added. After being stirred at 100 °C for 20 h, the reaction mixture was cooled to room temperature, diluted with water and dichloromethane, and the phases were separated. The aqueous phase was extracted twice with dichloromethane (20 mL), and the combined organic phases were washed with brine (1 × 20 mL). The organic phases were dried over Na_2_SO_4_ and filtered. The filtrate was concentrated to afford the crude product. Purification by column chromatography on silica gel (12% EtOAc in hexanes) afforded a yellow solid (665.7 mg, 3.41 mmol, 72%). ^1^H NMR(CDCl_3_, 400 MHz): δ (ppm) 7.57–7.50 (m, 2H), 7.47 (dd, *J* = 7.7, 1.5 Hz, 1H), 7.38–7.32 (m, 2H), 7.28–7.23 (m, 2H), 7.15 (td, *J* = 7.6, 1.6 Hz, 1H), 7.03 (d, *J* = 16.0 Hz, 1H), 6.92 (td, *J* = 7.5, 1.2 Hz, 1H), 6.87 (dd, *J* = 7.9, 1.2 Hz, 1H), 5.08 (s, 1H). ^13^C NMR(CDCl3, 101 MHz): δ (ppm) 141.67, 137.45, 130.92, 128.67, 127.66, 127.23, 126.55, 125.12, 123.82, 120.62, 117.38.

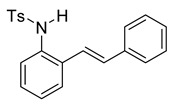

  *(E)-4-methyl-N-(2-styrylphenyl)benzenesulfonamide* (**10a**). To a solution of **9** (0.45 g, 2.28 mmol, 1.0 equiv) and pyridine (0.55 mL, 6.8 mmol, 3.0 equiv) in DCM (23 mL, 0.25 M), we added *p*-toluenesulfonyl chloride (0.52 g, 2.7 mmol, 1.1 equiv.) at 0 °C. The reaction was stirred in an ice bath while warming to room temperature for 12 h. The reaction mixture was quenched by the addition of a saturated aqueous solution of NH_4_Cl, and then, the product was extracted with DCM (10 mL × 3). The combined organic phase was washed with brine, dried over Na_2_SO_4_, filtered, and concentrated under reduced pressure. The residue was purified via flash column chromatography on silica gel (20% EtOAc in hexanes) to afford a white solid (749.3 mg, 2.17 mmol, 95%). ^1^H NMR(CDCl_3_, 400 MHz): δ (ppm) 7.57–7.50 (m, 2H), 7.46–7.37 (m, 1H), 7.34–7.19 (m, 6H), 7.18–7.12 (m, 2H), 7.12–7.06 (m, 2H), 6.71 (s, 2H), 6.39 (s, 1H), 2.23 (s, 3H). ^13^C NMR(CDCl_3_, 101 MHz): δ (ppm) 143.95, 136.65, 136.57, 133.22, 133.17, 132.34, 129.68, 128.65, 128.45, 128.15, 127.17, 127.08, 126.72, 126.64, 122.60, 21.49.

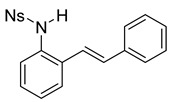

  *(E)-4-nitro-N-(2-styrylphenyl)benzenesulfonamide* (**10b**). To a solution of **9** (0.165 g, 0.845 mmol, 1.0 equiv.) and pyridine (0.20 mL, 2.53 mmol, 3.0 equiv) in DCM (8.5 mL, 0.25 M), we added 4-nitrobenzenesulfonyl chloride (0.22 g, 1.01 mmol, 1.1 equiv) at 0 °C. The reaction was stirred in an ice bath while warming to room temperature for 12 h. The reaction mixture was quenched by the addition of a saturated aqueous solution of NH_4_Cl, and then, the product was extracted with DCM (10 mL × 3). The combined organic phase was washed with brine, dried over Na_2_SO_4_, filtered, and concentrated under reduced pressure. The residue was purified via flash column chromatography on silica gel (20% EtOAc in hexanes) to afford a yellow solid (268.7 mg, 0.71 mmol, 84%). 1H NMR(CDCl_3_, 400 MHz): δ (ppm) 8.18–8.13 (m, 2H), 7.90–7.86 (m, 2H), 7.56–7.51 (m, 1H), 7.41–7.31 (m, 6H), 7.28–7.24 (m, 2H), 6.76 (d, *J* = 3.0 Hz, 2H), 6.62 (s, 1H). ^13^C NMR(CDCl_3_, 101 MHz): δ (ppm) 150.10, 145.03, 136.19, 134.02, 132.78, 131.91, 128.85, 128.73, 128.58, 128.45, 128.26, 127.74, 126.85, 126.44, 124.18, 122.12.

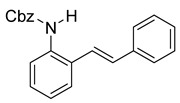

  *Benzyl (E)-(2-styrylphenyl)carbamate* (**10c**). To a solution of **9** (0.24 g, 1.23 mmol, 1.0 equiv) and pyridine (0.30 mL, 3.7 mmol, 3.0 equiv) in DCM (4.1 mL, 0.25 M), we added benzyl chloroformate (0.21 mL, 1.5 mmol, 1.2 equiv) at 0 °C. The reaction was stirred in an ice bath while warming to room temperature for 12 h. The reaction mixture was quenched by the addition of a saturated aqueous solution of NH_4_Cl, and then, the product was extracted with DCM (3 × 10 mL). The combined organic phase was washed with brine, dried over Na_2_SO_4_, filtered, and concentrated under reduced pressure. The residue was purified via flash column chromatography on silica gel (20% EtOAc in hexanes) to afford an off-white solid (397 mg, 1.21 mmol, 98%).

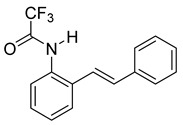

  *(E)-2,2,2-trifluoro-N-(2-styrylphenyl)acetamide* (**10d**). To a solution of **9** (0.33 g, 1.70 mmol, 1.0 equiv) and pyridine (0.41 mL, 5.1 mmol, 3.0 equiv) in DCM (5.7 mL, 0.25 M), we added TFAA (0.28 mL, 1.2 equiv) at 0 °C. The reaction was stirred in an ice bath while warming to room temperature for 12 h. The reaction mixture was quenched by the addition of a saturated aqueous solution of NH_4_Cl, and then, the product was extracted with DCM (3 × 10 mL). The combined organic phase was washed with brine, dried over Na_2_SO_4_, filtered, and concentrated under reduced pressure to afford a white solid that was used without further purification (450.5 mg, 1.55 mmol, 91%). ^1^H NMR(CDCl_3_, 400 MHz): δ (ppm) 7.86 (s, 1H), 7.79 (dd, *J* = 8.0, 1.5 Hz, 1H), 7.52 (dd, *J* = 7.6, 1.7 Hz, 1H), 7.46–7.41 (m, 2H), 7.36–7.21 (m, 5H), 7.06–6.92 (m, 2H). ^13^C NMR(CDCl_3_, 101 MHz): δ (ppm) 155.13 (q, *J* = 36.8 Hz), 136.38, 134.25, 131.63, 130.87, 128.92, 128.61, 128.57, 127.54, 127.30, 126.74, 123.78, 121.93, 115.13 (q, *J* = 288.2 Hz).

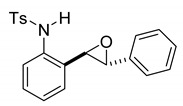

  *rac-4-methyl-N-(2-(3-phenyloxiran-2-yl)phenyl)benzenesulfonamide* (**11a**). To a solution of **10a** (175 mg, 0.5 mmol) in dichloromethane (10 mL), we added NaHCO_3_ (84 mg, 1.0 mmol.). The solution was stirred under a stream of nitrogen followed by the addition of *m*CPBA (70–75%, 247 mg, 1.0 mmol.). The reaction was allowed to stir for 3 h. TLC (20% EA in hexanes) indicated complete conversion. The reaction was quenched by the addition of 10 mL of a sat’d NaHCO_3_ solution followed by 10 mL of a sat’d NaHSO_3_ solution. This suspension was allowed to stir for 5 min before being poured into a separatory funnel, extracted with dichloromethane (3 × 10 mL), dried over Na_2_SO_4_, and concentrated to give an off-white solid that was used without further purification (179.9 mg, 0.49 mmol, 98%). A small sample of this crude material was analyzed with NMR to determine if epoxide was formed. ^1^H NMR(CDCl_3_, 400 MHz): δ (ppm) 7.64 (s, 1H), 7.62–7.55 (m, 2H), 7.37–7.27 (m, 4H), 7.18–7.04 (m, 7H), 3.68 (d, *J* = 2.1 Hz, 1H), 3.65 (d, *J* = 2.2 Hz, 1H), 2.33 (s, 3H). ^13^C NMR(CDCl_3_, 101 MHz): δ (ppm) 143.90, 136.58, 135.79, 135.14, 129.70, 128.94, 128.74, 128.67, 127.82, 127.55, 127.25, 125.57, 123.68, 62.06, 60.64, 21.59.

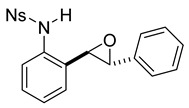

  *rac-4-nitro-N-(2-(3-phenyloxiran-2-yl)phenyl)benzenesulfonamide* (**11b**). To a solution of **10b** (186 mg, 0.49 mmol) in dichloromethane (10 mL), we added NaHCO_3_ (82 mg, 0.98 mmol). The solution was stirred under a stream of nitrogen followed by the addition of mCPBA (70–75%, 241 mg). The reaction was allowed to stir for 3 h. TLC (20% EA in hexanes) indicated complete conversion. The reaction was quenched by the addition of 10 mL of a sat’d NaHCO_3_ solution followed by 10 mL of a sat’d NaHSO_3_ solution. This suspension was allowed to stir for 5 min before being poured into a separatory funnel, extracted with dichloromethane (3 × 10 mL), dried over Na_2_SO_4_, and concentrated. The crude yellow solid was used in the next step without any further purification.

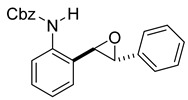

  *rac-benzyl (2-((2R,3R)-3-phenyloxiran-2-yl)phenyl)carbamate* (**11c**). To a solution of **10c** (114 mg, 0.34 mmol) in dichloromethane (7 mL), we added NaHCO_3_ (58 mg, 0.69 mmol.). The solution was stirred under a stream of nitrogen followed by the addition of *m*CPBA (70–75%, 170 mg, 0.69 mmol.). The reaction was allowed to stir for 18 h. TLC (10% EA in hexanes) indicated complete conversion. The reaction was quenched by the addition of 10 mL of a sat’d NaHCO_3_ solution followed by 10 mL of a sat’d NaHSO_3_ solution. This suspension was allowed to stir for 5 min before being poured into a separatory funnel, extracted with dichloromethane (3 × 10 mL), dried over Na_2_SO_4_, and concentrated to provide an off-white solid that was used without further purification (114 mg, 0.33 mmol, 96%). A small sample of this crude material was analyzed with NMR to determine if epoxide was formed. ^1^H NMR(CDCl_3_, 400 MHz): δ (ppm) 7.97–7.73 (m, 2H), 7.35–7.19 (m, 11H), 7.03 (td, *J* = 7.5, 1.2 Hz, 1H), 5.14–5.04 (m, 2H), 4.00 (d, *J* = 2.2 Hz, 1H), 3.91 (d, *J* = 2.1 Hz, 1H). ^13^C NMR(CDCl_3_, 101 MHz): δ (ppm) 153.77, 136.40, 136.20, 136.11, 128.98, 128.69, 128.63, 128.60, 128.58, 128.34, 128.31, 128.29, 127.53, 125.59, 67.03, 62.72, 60.15.

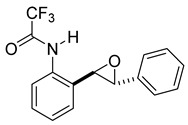

  *rac-2,2,2-trifluoro-N-(2-(3-phenyloxiran-2-yl)phenyl)acetamide* (**11d**). To a solution of **10d** (322 mg, 1.1 mmol) in dichloromethane (22 mL), we added NaHCO_3_ (186 mg, 2.2 mmol.). The solution was stirred under a stream of nitrogen followed by the addition of *m*CPBA (70–75%, 545 mg, 2.2 mmol.). The reaction was allowed to stir for 18 h. TLC (10% EA in hexanes) indicated complete conversion. The reaction was quenched by the addition of 20 mL of a sat’d NaHCO_3_ solution followed by 20 mL of a sat’d NaHSO_3_ solution. This suspension was allowed to stir for 5 min before being poured into a separatory funnel, extracted with dichloromethane (3 × 10 mL), dried over Na_2_SO_4_, and concentrated to provide a gum that was used without further purification (1.1 mmol, 343 mg, quantitative). A small sample of this crude material was analyzed with NMR to determine if epoxide was formed. ^1^H NMR(CDCl_3_, 400 MHz): δ (ppm) 10.21 (s, 1H), 8.24 (dd, *J* = 8.3, 1.1 Hz, 1H), 7.39–7.23 (m, 7H), 7.15 (td, *J* = 7.6, 1.2 Hz, 1H), 4.08 (d, *J* = 2.3 Hz, 1H), 4.03 (d, *J* = 2.2 Hz, 1H). ^13^C NMR(CDCl_3_, 101 MHz): δ (ppm) 154.93 (q, *J* = 40.0 Hz), 135.10, 134.23, 129.28, 129.06, 128.84, 128.62, 125.74, 125.58, 124.15, 121.97, 115.82 (q, *J* = 292.7 Hz), 64.11, 60.28.

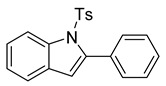

  *2-phenyl-1-tosyl-1H-indole* (**12a**). To a stirred solution of **11a** (in DCM (1.0 mL), we added TFA (0.1 mL). The solution immediately changed into a darker color. TLC (20% EtOAc in hexanes) indicated the complete conversion of the crude epoxide into indole. The solution was poured into 10 mL of sat’d NaHCO_3_ followed by the addition of 10 mL of DCM. The layers were poured into a separatory funnel. The organic layer was collected, and the aqueous layer was extracted with DCM (2 × 15 mL). The combined organic layers were dried over Na_2_SO_4_, filtered, and concentrated to provide a light yellow solid. ^1^H NMR(CDCl_3_, 400 MHz): δ (ppm) 8.24 (dd, *J* = 8.4, 0.9 Hz, 1H), 7.44–7.40 (m, 2H), 7.39–7.33 (m, 4H), 7.28 (ddd, *J* = 8.5, 7.3, 1.4 Hz, 2H), 7.21–7.18 (m, 3H), 6.99–6.92 (m, 2H), 6.47 (d, *J* = 0.8 Hz, 1H), 2.21 (s, 3H). ^13^C NMR(CDCl_3_, 101 MHz): δ (ppm) 144.52, 142.13, 138.27, 134.66, 132.42, 130.55, 130.33, 129.20, 128.65, 127.50, 126.80, 124.78, 124.30, 120.68, 116.66, 113.62, 21.53.

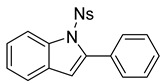

  *1-((4-nitrophenyl)sulfonyl)-2-phenyl-1H-indole* (**12b**) [To a stirred solution of **11b** (193 mg, 0.49 mmol) in DCM (10 mL), we added TFA (1 mL). The solution immediately changed into a darker color. After 18 h, TLC (20% EtOAc in hexanes) indicated the partial conversion of the crude epoxide into indole. The solution was poured into 10 mL of sat’d NaHCO_3_ followed by the addition of 10 mL of DCM. The layers were poured into a separatory funnel. The organic layer was collected, and the aqueous layer was extracted with DCM (2 × 15 mL). The combined organic layers were dried over Na_2_SO_4_, filtered, and concentrated to provide a dark yellow oil. This residue was purified via column chromatography (20% EtOAc in hexanes) to provide a yellow solid (87 mg, 0.23 mmol, 48%). ^1^H NMR(CDCl_3_, 400 MHz): δ (ppm) 8.22 (dq, *J* = 8.4, 0.9 Hz, 1H), 8.04–8.00 (m, 2H), 7.48–7.37 (m, 8H), 7.33 (ddd, *J* = 8.5, 7.3, 1.4 Hz, 1H), 7.24 (td, *J* = 7.5, 1.0 Hz, 1H), 6.53 (d, *J* = 0.8 Hz, 1H). ^13^C NMR(CDCl_3_, 101 MHz): δ (ppm) 150.44, 142.27, 141.94, 138.08, 131.72, 130.77, 130.18, 129.12, 128.09, 127.82, 125.44, 125.20, 123.79, 121.17, 116.72, 114.79.

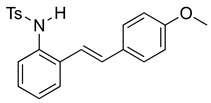

  *(E)-N-(2-(4-methoxystyryl)phenyl)benzenesulfonamide* (**10e**). To a solution of 2-iodoaniline (1.00 g, 4.58 mmol, 1.0 eq.) in triethylamine (4.6 mL, 1.0 M), Pd(OAc)_2_ (10.2 mg, 0.046 mmol, 1 mol%), P(*o*-Tol)_3_ (111 mg, 0.36 mmol, 8 mol%), and 4-methoxystyrene (735 mg, 5.48 mmol, 1.2 eq.) were added. After being stirred at 100 °C for 20 h, the reaction mixture was cooled to room temperature, diluted with water and dichloromethane, and the phases were separated. The aqueous phase was extracted twice with dichloromethane (20 mL), and the combined organic phases were washed with brine (1 × 20 mL). The organic phases were dried over Na_2_SO_4_ and filtered. The filtrate was concentrated to afford the crude product. Purification via column chromatography on silica gel (12% EtOAc in hexanes) afforded a white solid (733 mg, 3.25 mmol, 71%). NMR spectra were consistent with data reported in the literature [7]. To a solution of this styrylaniline (370 mg, 1.64 mmol) and pyridine (3.0 equiv) in DCM (0.25 M), we added *p*-toluenesulfonyl chloride (1.1 equiv, 1.97 mmol, 356 mg) at 0 °C. The reaction was stirred in an ice bath while warming to room temperature for 12 h. The reaction mixture was quenched by the addition of a saturated aqueous solution of NH_4_Cl, and then, the product was extracted with DCM (3 × 10 mL). The combined organic phase was washed with brine, dried over Na_2_SO_4_, filtered, and concentrated under reduced pressure. The residue was purified via flash column chromatography on silica gel (20% EtOAc in hexanes) to afford a white solid (453 mg, 1.19 mmol, 73%). NMR spectra were consistent with data reported in the literature [7].

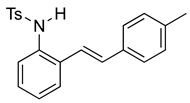

  *(E)-4-Methyl-N-(2-(4-methylstyryl)phenyl)benzenesulfonamide* (**10f**). To a solution of 2-iodoaniline (1.00 g, 4.58 mmol, 1.0 eq.) in triethylamine (4.6 mL, 1.0 M), Pd(OAc)_2_ (10.3 mg, 0.046 mmol, 1 mol%), P(*o*-Tol)_3_ (111 mg, 0.37 mmol, 8 mol%), and 4-methylstyrene (762 mg, 5.5 mmol, 1.2 eq.) were added. After being stirred at 100 °C for 20 h, the reaction mixture was cooled to room temperature, diluted with water and dichloromethane, and the phases were separated. The aqueous phase was extracted twice with dichloromethane (20 mL), and the combined organic phases were washed with brine (1 × 20 mL). The organic phases were dried over Na_2_SO_4_ and filtered. The filtrate was concentrated to afford the crude product. Purification via column chromatography on silica gel (12% EtOAc in hexanes) afforded a white solid (627 mg, 3.00 mmol, 65%). NMR spectra were consistent with data reported in the literature [7]. To a solution of this styrylaniline (611 mg, 2.91 mmol) and pyridine (0.70 mL, 8.76 mmol, 3.0 equiv) in DCM (12 mL, 0.25 M), we added *p*-toluenesulfonyl chloride (668 mg, 3.5 mmol, 1.2 equiv.) at 0 °C. The reaction was stirred in an ice bath while warming to room temperature for 12 h. The reaction mixture was quenched by the addition of a saturated aqueous solution of NH_4_Cl, and then, the product was extracted with DCM (3 × 10 mL). The combined organic phase was washed with brine, dried over Na_2_SO_4_, filtered, and concentrated under reduced pressure. The residue was purified via flash column chromatography on silica gel (20% EtOAc in hexanes) to afford a white solid (788 mg, 2.17 mmol, 74%). ^1^H NMR(CDCl_3_, 400 MHz): δ (ppm) 7.54 (d, *J* = 8.3 Hz, 2H), 7.35 (ddd, *J* = 32.3, 7.0, 2.6 Hz, 2H), 7.18–7.03 (m, 8H), 6.72–6.58 (m, 2H), 6.36 (s, 1H), 2.30 (s, 3H), 2.24 (s, 3H). ^13^C NMR(CDCl3, 101 MHz): δ (ppm) 143.92, 138.18, 136.60, 133.88, 133.20, 133.14, 132.40, 129.67, 129.37, 128.26, 127.16, 126.98, 126.61, 126.56, 126.50, 121.49, 21.50, 21.30.

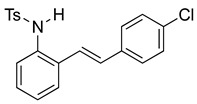

  *(E)-N-(2-(4-chlorostyryl)phenyl)benzenesulfonamide* (**10g**). To a solution of 2-iodoaniline (1.00 g, 4.58 mmol, 1.0 eq.) in triethylamine (4.6 mL, 1.0 M), Pd(OAc)_2_ (10.3 mg, 0.046 mmol, 1 mol%), P(*o*-Tol)_3_ (111 mg, 0.37 mmol, 8 mol%), and 4-chlorostyrene (760 mg, 5.5 mmol, 1.2 eq.) were added. After being stirred at 100 °C for 20 h, the reaction mixture was cooled to room temperature, diluted with water and dichloromethane, and the phases were separated. The aqueous phase was extracted twice with dichloromethane (20 mL), and the combined organic phases were washed with brine (1 × 20 mL). The organic phases were dried over Na_2_SO_4_ and filtered. The filtrate was concentrated to afford the crude product. Purification via column chromatography on silica gel (12% EtOAc in hexanes) afforded a white solid (817.5 mg, 3.56 mmol, 77%). NMR spectra were consistent with data reported in the literature [7]. To a solution of this styrylaniline (363 mg, 1.58 mmol) and pyridine (0.38 mL, 4.74 mmol, 3.0 equiv) in DCM (5.2 mL, 0.25 M), we added *p*-toluenesulfonyl chloride (362 mg, 1.9 mmol, 1.2 equiv) at 0 °C. The reaction was stirred in an ice bath while warming to room temperature for 12 h. The reaction mixture was quenched by the addition of a saturated aqueous solution of NH_4_Cl, and then, the product was extracted with DCM (10 mL × 3). The combined organic phase was washed with brine, dried over Na_2_SO_4_, filtered, and concentrated under reduced pressure. The residue was purified via flash column chromatography on silica gel (20% EtOAc in hexanes) to afford a white solid (532 mg, 1.39 mmol, 88%). NMR spectra were consistent with data reported in the literature [7].

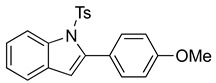

  *N-Ts-2-(4-methoxyphenyl)indole* (**12c**). To a solution of **10e** (127 mg, 0.33 mmol)) in DCM (7 mL), we added NaHCO_3_ (54 mg, 0.64 mmol, 2 equiv). The solution was stirred under a stream of nitrogen followed by the addition of mCPBA (70%, 154 mg, 0.62 mmol, 2 equiv.). The reaction was allowed to stir for 3 h. TLC (20% EA in hexanes) indicated complete conversion. The reaction was quenched by the addition of 10 mL of a sat’d NaHCO_3_ solution followed by 10 mL of a sat’d NaHSO_3_ solution. This suspension was allowed to stir for 5 min before being poured into a separatory funnel, extracted with DCM (3 × 10 mL), dried over Na_2_SO_4_, and concentrated. The residue was dissolved in DCM (6.6 mL) with stirring. To this stirred solution, we added TFA (~0.7 mL), which was followed by an immediate color change. TLC (20% EA in hexanes), which indicated the complete conversion of the crude epoxide into indole. The reaction was poured into a separatory funnel and washed twice with 10 mL of a sat’d NaHSO_3_ solution. The organic layer was dried over Na_2_SO_4_ and filtered. The filtrate was concentrated to afford a light yellow solid (99.1 mg, 0.26 mmol, 80%). NMR spectra were consistent with data reported in the literature [7].

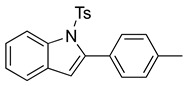

  *N-Ts-2-(4-methylphenyl)indole* (**12d**). To a solution of **10f** (45 mg, 0.13 mmol) in DCM (3 mL), we added NaHCO_3_ (21 mg, 0.28 mmol, 2 equiv.). The solution was stirred under a stream of nitrogen followed by the addition of mCPBA (70%, 62 mg, 0.28 mmol, 2 equiv.). The reaction was allowed to stir for 3 h. TLC (20% EA in hexanes) indicated complete conversion. The reaction was quenched by the addition of 10 mL of a sat’d NaHCO_3_ solution followed by 10 mL of a sat’d NaHSO_3_ solution. This suspension was allowed to stir for 5 min before being poured into a separatory funnel, extracted with DCM (3 × 10 mL), dried over Na_2_SO_4_, and concentrated. The residue was dissolved in DCM (2.6 mL) with stirring. To this stirred solution, we added TFA (~0.3 mL), which was followed by an immediate color change. TLC (20% EA in hexanes) indicated the complete conversion of the crude epoxide into indole. The reaction was poured into a separatory funnel and washed twice with 10 mL of a sat’d NaHSO_3_ solution. The organic layer was dried over Na_2_SO_4_ and filtered. The filtrate was concentrated to afford a light yellow solid (36 mg, 0.10 mmol, 78%). NMR spectra were consistent with data reported in the literature [7].

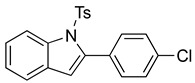

  *N-Ts-2-(4-chlorophenyl)indole* (**12e**). To a solution of **10g** (149 mg, 0.39 mmol)) in DCM (8 mL), we added NaHCO_3_ (66 mg, 0.79 mmol, 2 equiv.). The solution was stirred under a stream of nitrogen followed by the addition of mCPBA (70%, 195 mg, 0.79 mmol, 2 equiv.). The reaction was allowed to stir for 3 h. TLC (20% EA in hexanes) indicated complete conversion. The reaction was quenched by the addition of 10 mL of a sat’d NaHCO_3_ solution followed by 10 mL of a sat’d NaHSO_3_ solution. This suspension was allowed to stir for 5 min before being poured into a separatory funnel, extracted with DCM (3 × 10 mL), dried over Na_2_SO_4_, and concentrated. The residue was dissolved in DCM (8 mL) with stirring. To this stirred solution, we added TFA (~0.8 mL), which was followed by an immediate color change. TLC (20% EA in hexanes) indicated the complete conversion of the crude epoxide into indole. The reaction was poured into a separatory funnel and washed twice with 10 mL of a sat’d NaHSO_3_ solution. The organic layer was dried over Na_2_SO_4_ and filtered. The filtrate was concentrated to afford a light yellow solid (34.6 mg, 0.30 mmol, 77%). NMR spectra were consistent with data reported in the literature [7].

## 4. Conclusions

In summary, we demonstrated an operationally simple and efficient method with which to synthesize 2-aryl-*N*-sulfonyl indoles (C1 substituted indoles) from 2-alkenylanilines. The chemical steps are few in number and are robust reactions that are tolerant of many functional groups. Our approach is novel in that it uses chemical reagents (*m*CPBA, NaHCO_3_, TFA, and CH_2_Cl_2_) that are relatively inexpensive and commonplace in synthetic laboratories. Additionally, the reagents are not air-sensitive, and the reactions are conducted at room temperature and under atmospheric pressure. It is our hope that this method will find utility in the synthesis of indoles and other heterocycles. Currently, our laboratory is investigating routes with fewer synthetic steps and routes that will furnish C2-substituted indoles.

## Data Availability

Data are contained within the article and Supplementary Materials.

## Supplementary Materials

The following supporting information can be downloaded at: https://www.mdpi.com/article/10.3390/molecules28247968/s1, Copies of NMR spectra.
