# Supplementary figures and images for: An Operationally Simple Approach to Indole Derivatives from 2-Alkenylanilines Utilizing an Oxidation–Intramolecular Cyclization–Elimination Sequence
Source: Molecules. 2023 Dec 6;28(24):7968. doi: 10.3390/molecules28247968 (PMC10745314; doi:10.3390/molecules28247968)

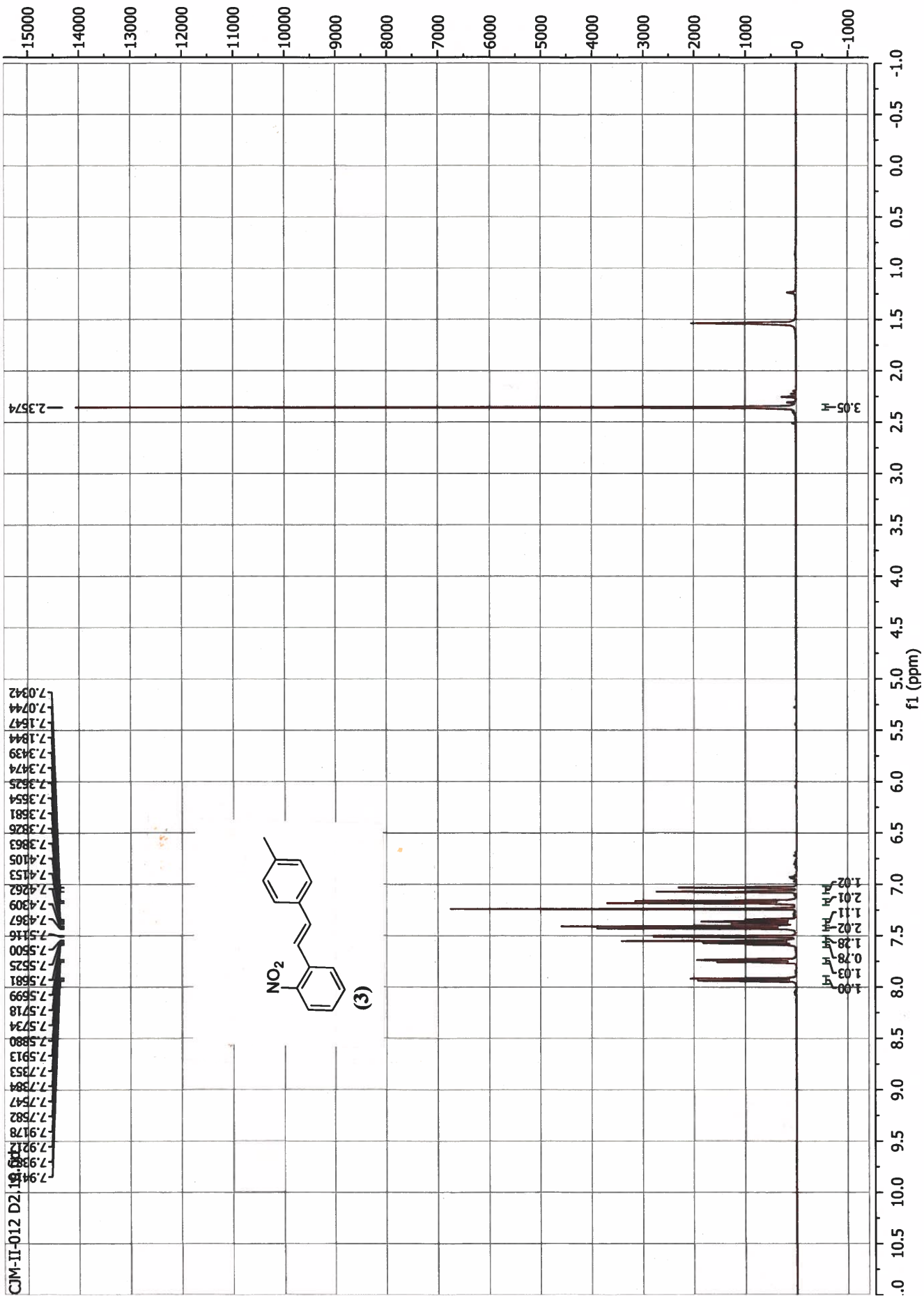

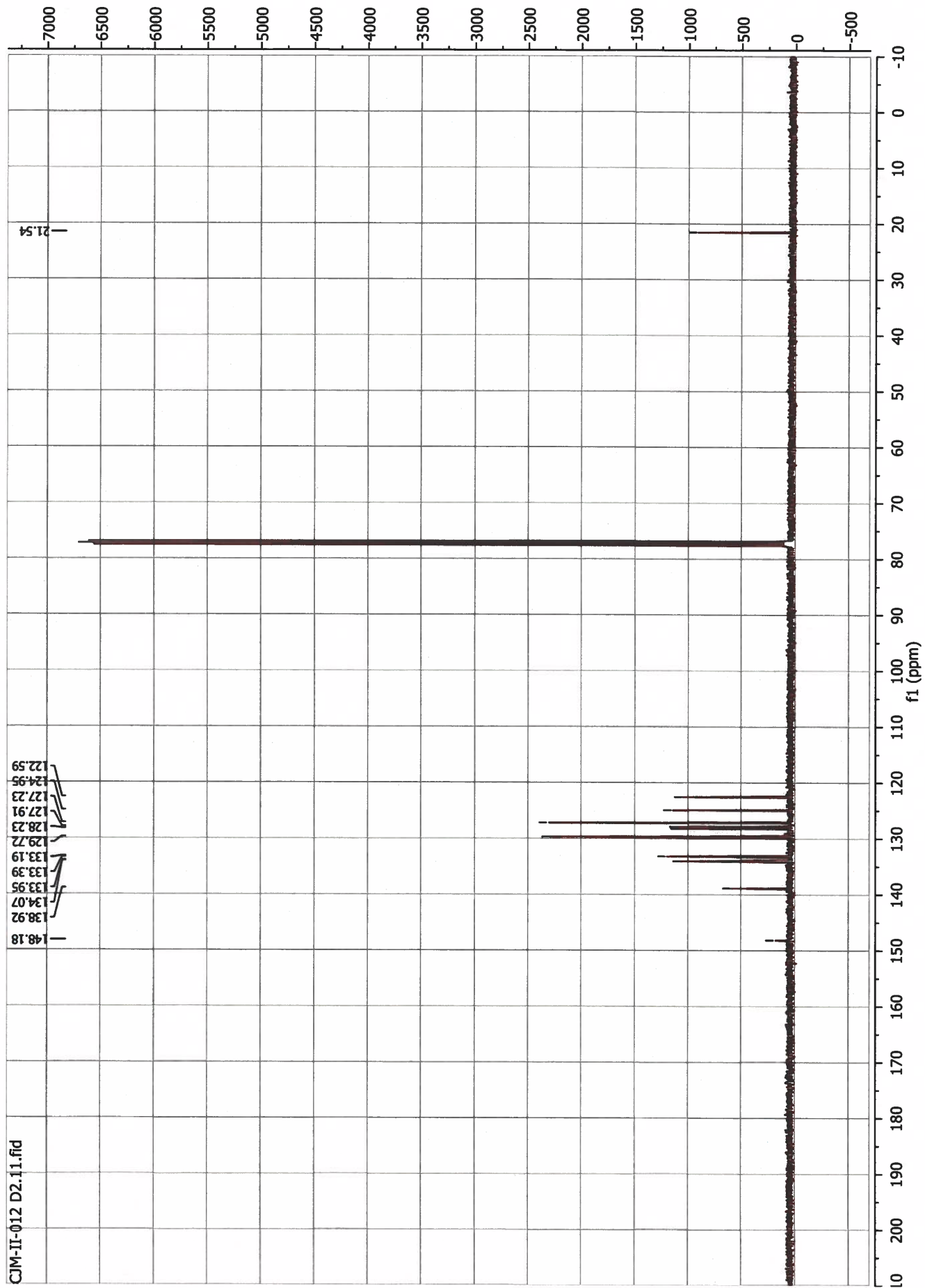

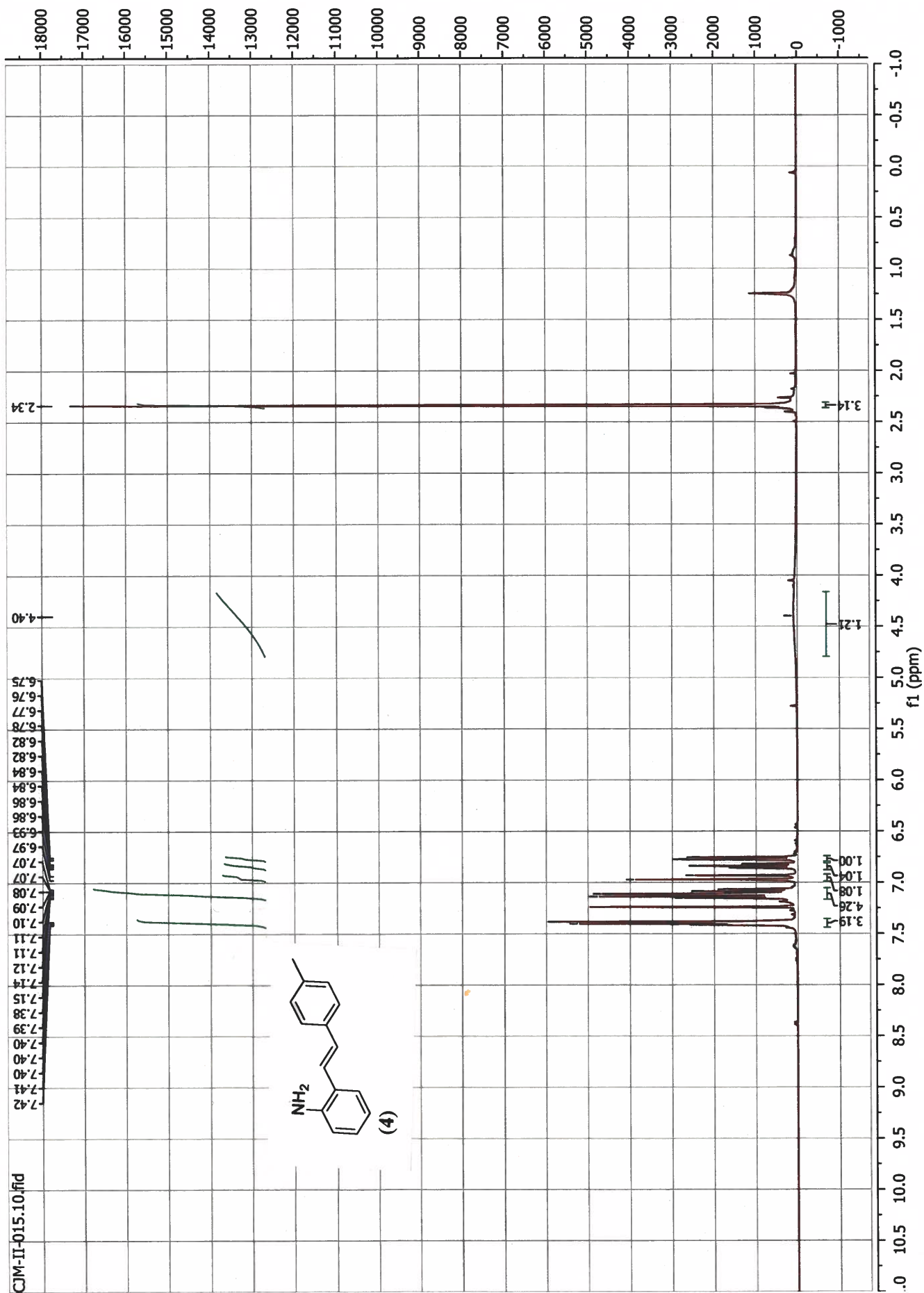

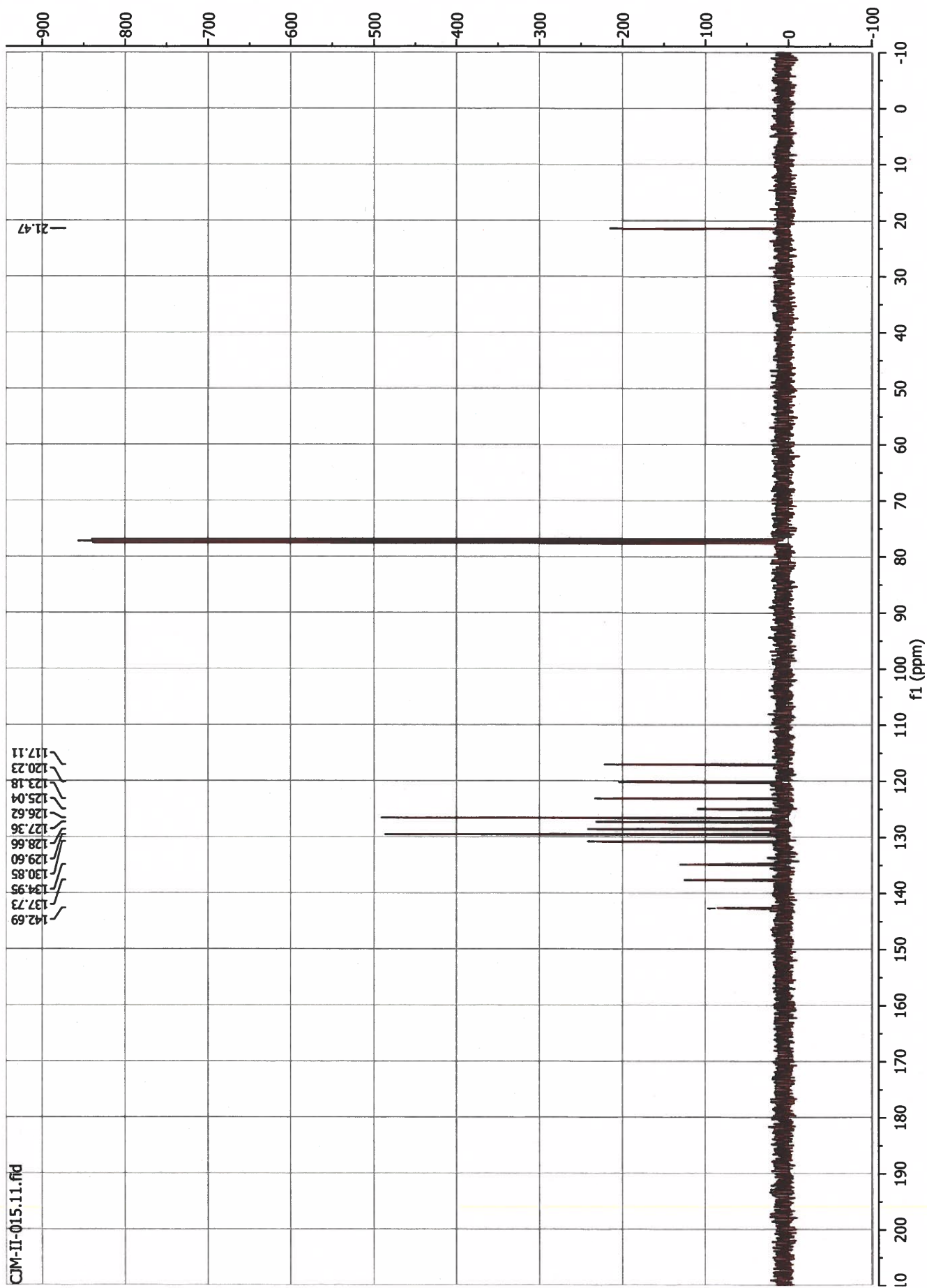

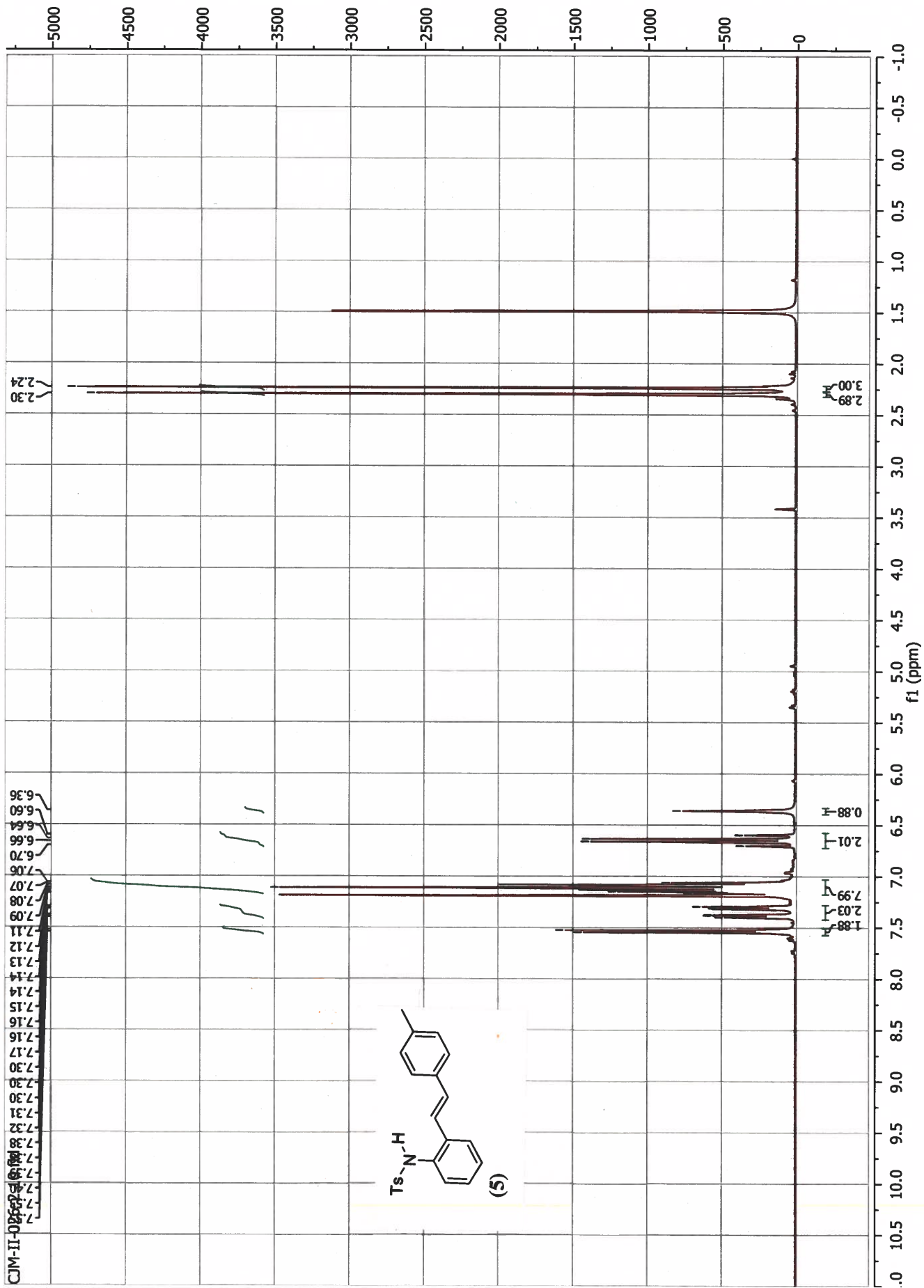

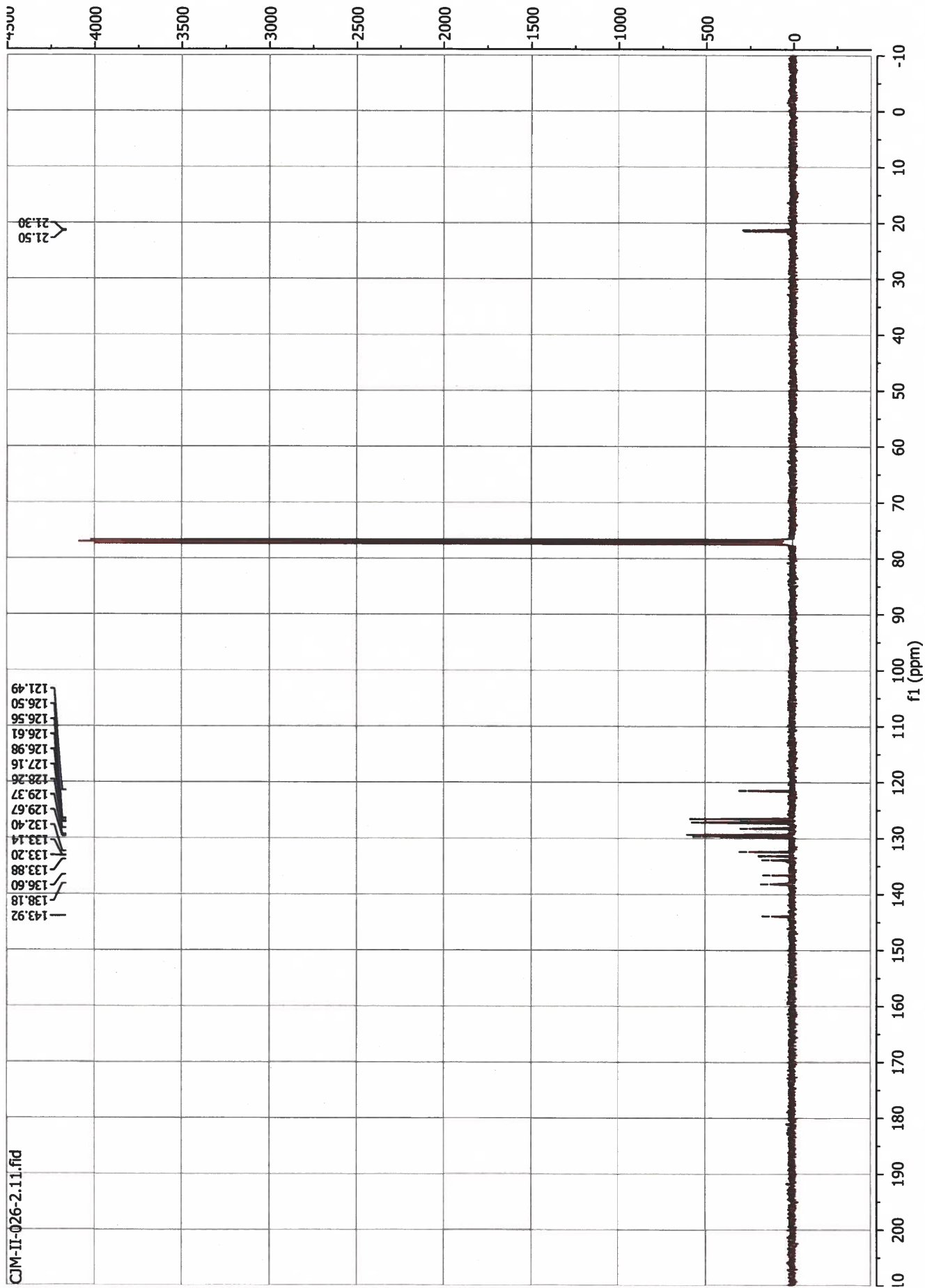

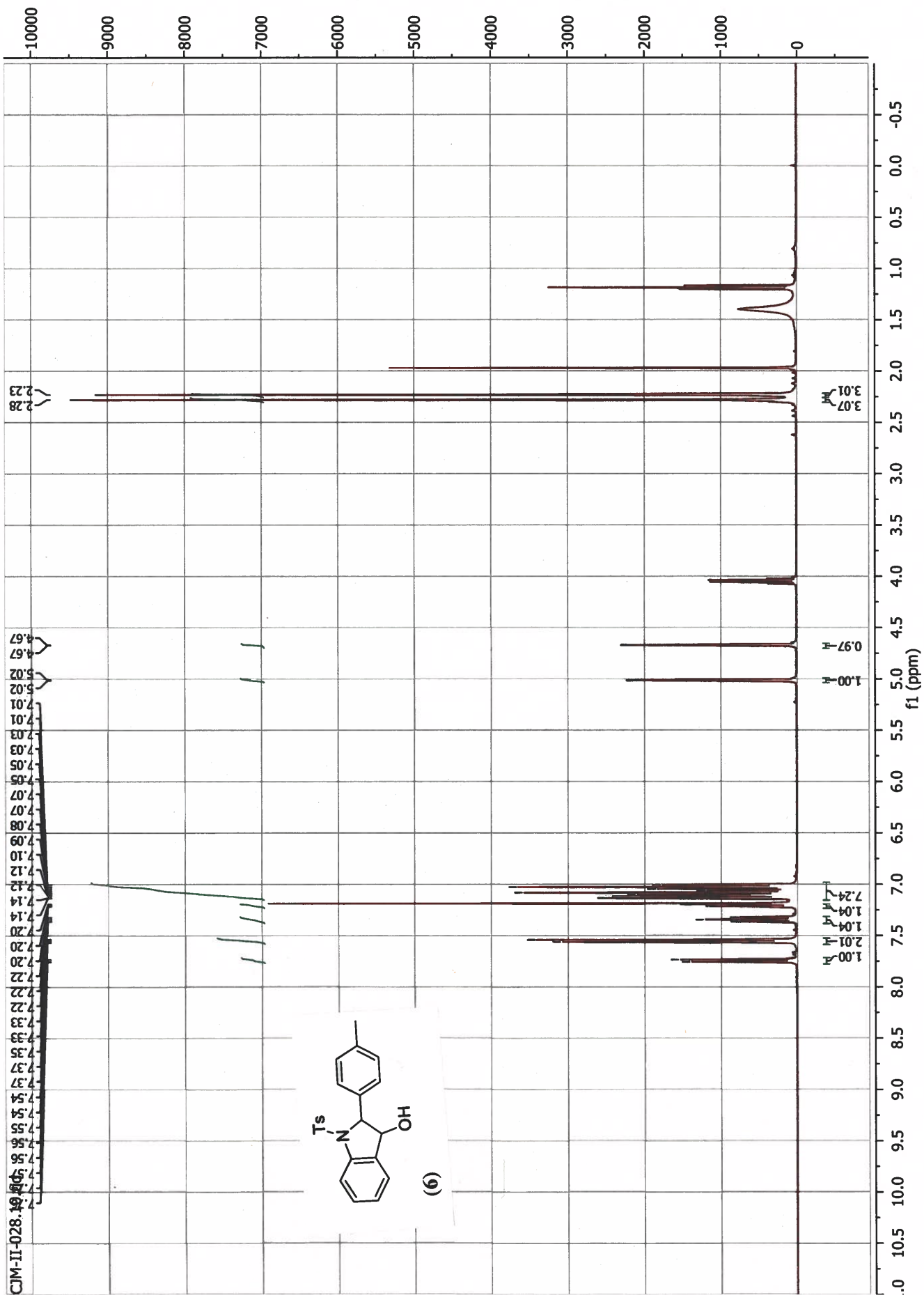

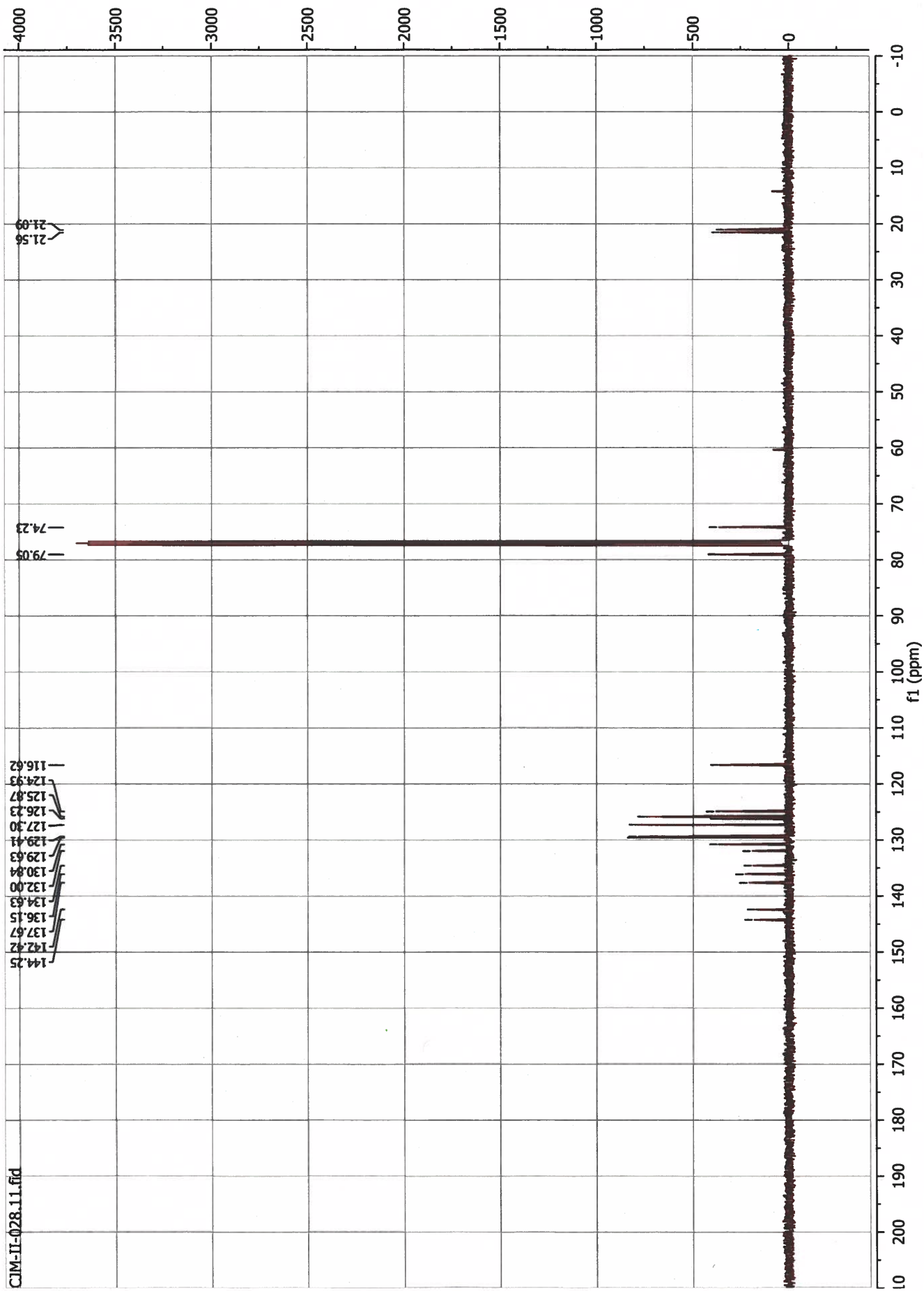

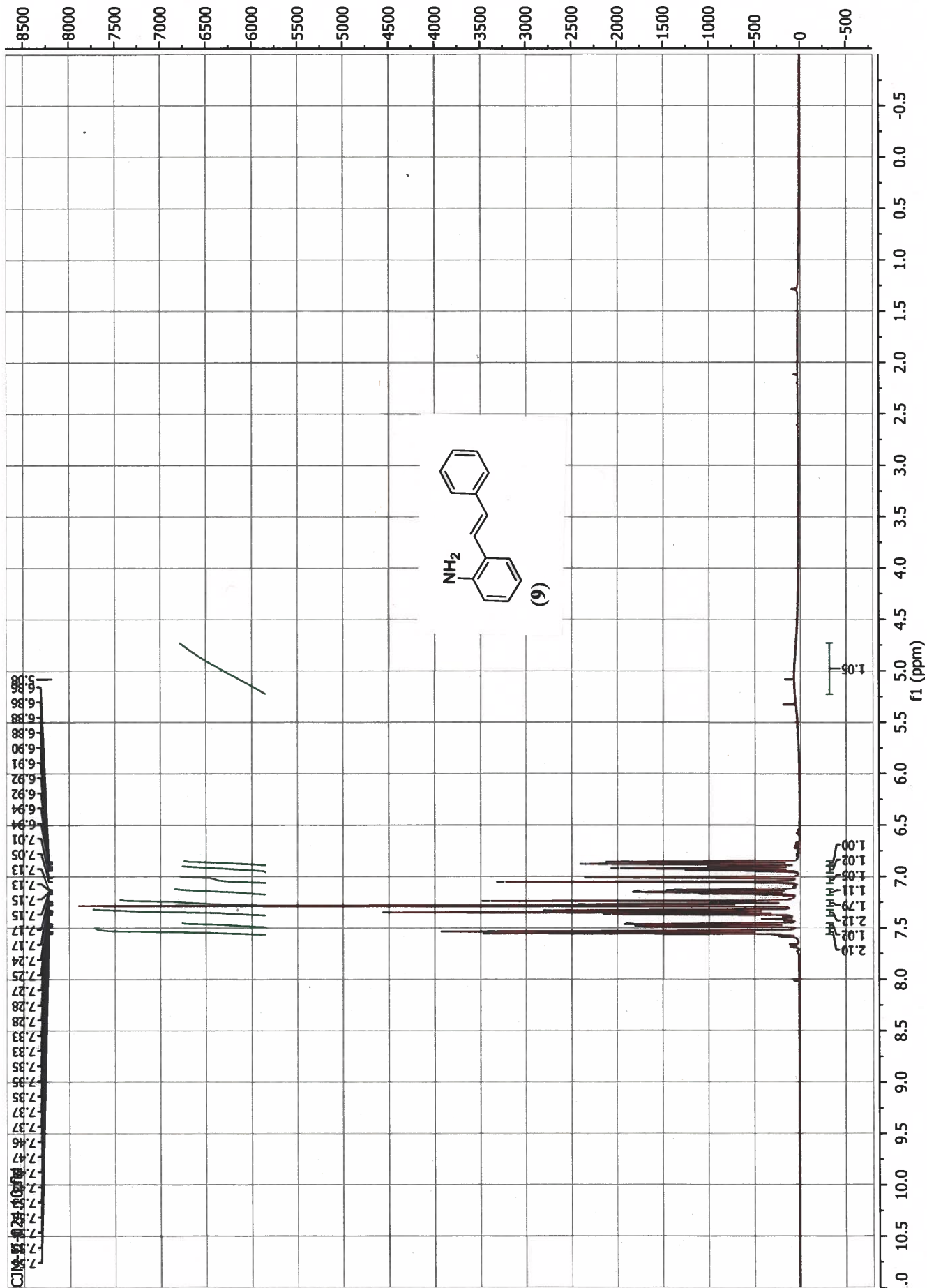

CJM-II-024.14.fid

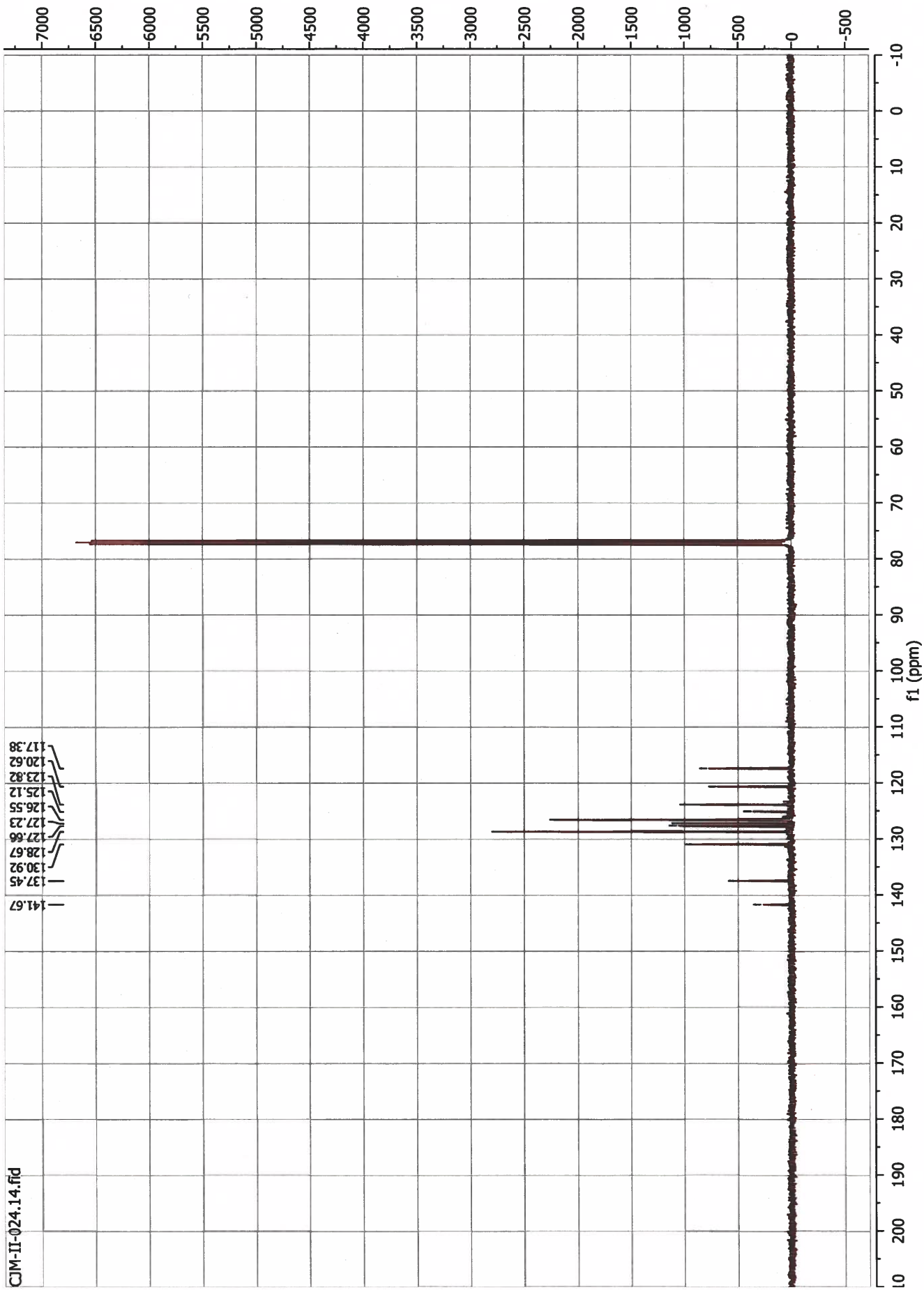

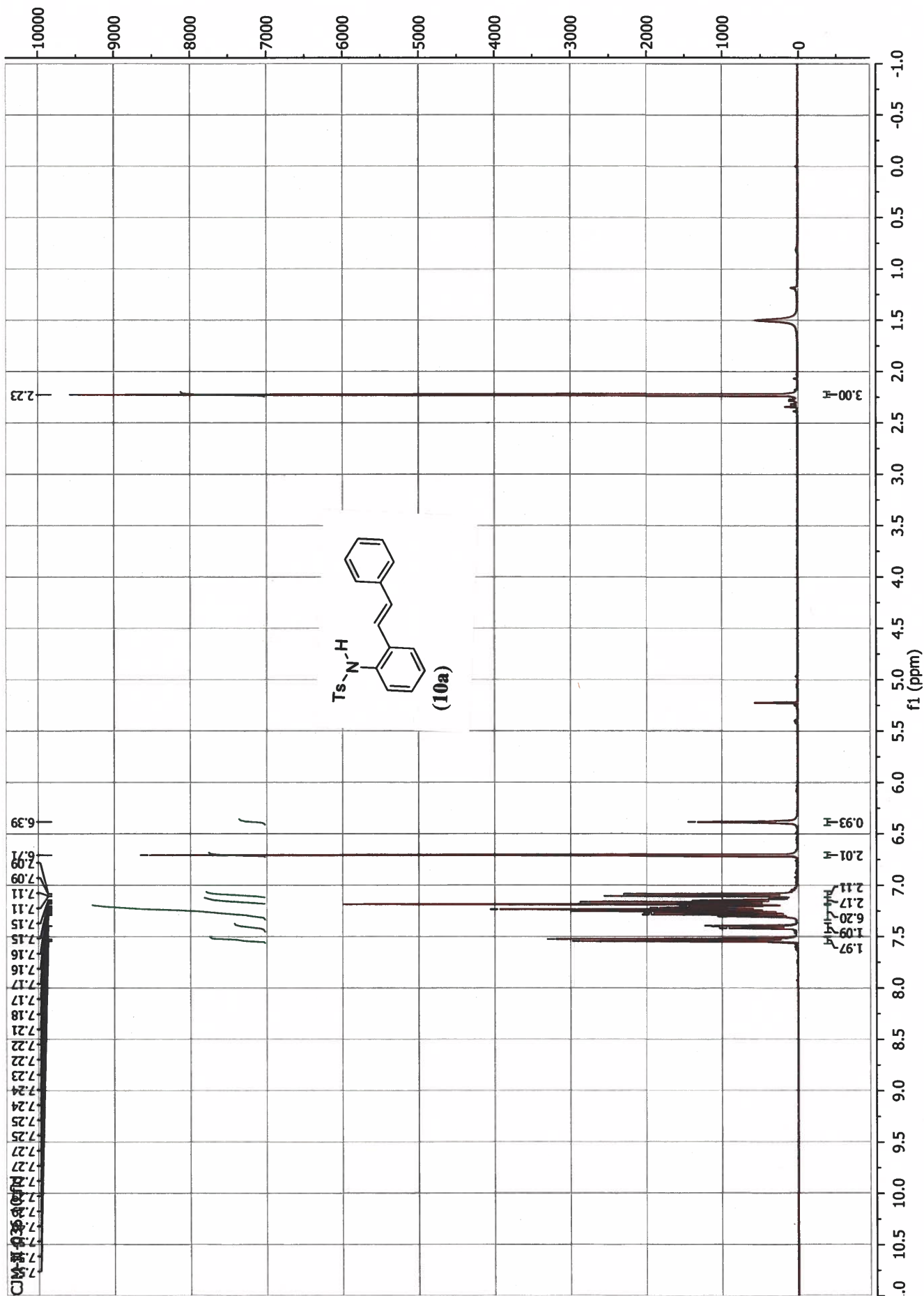

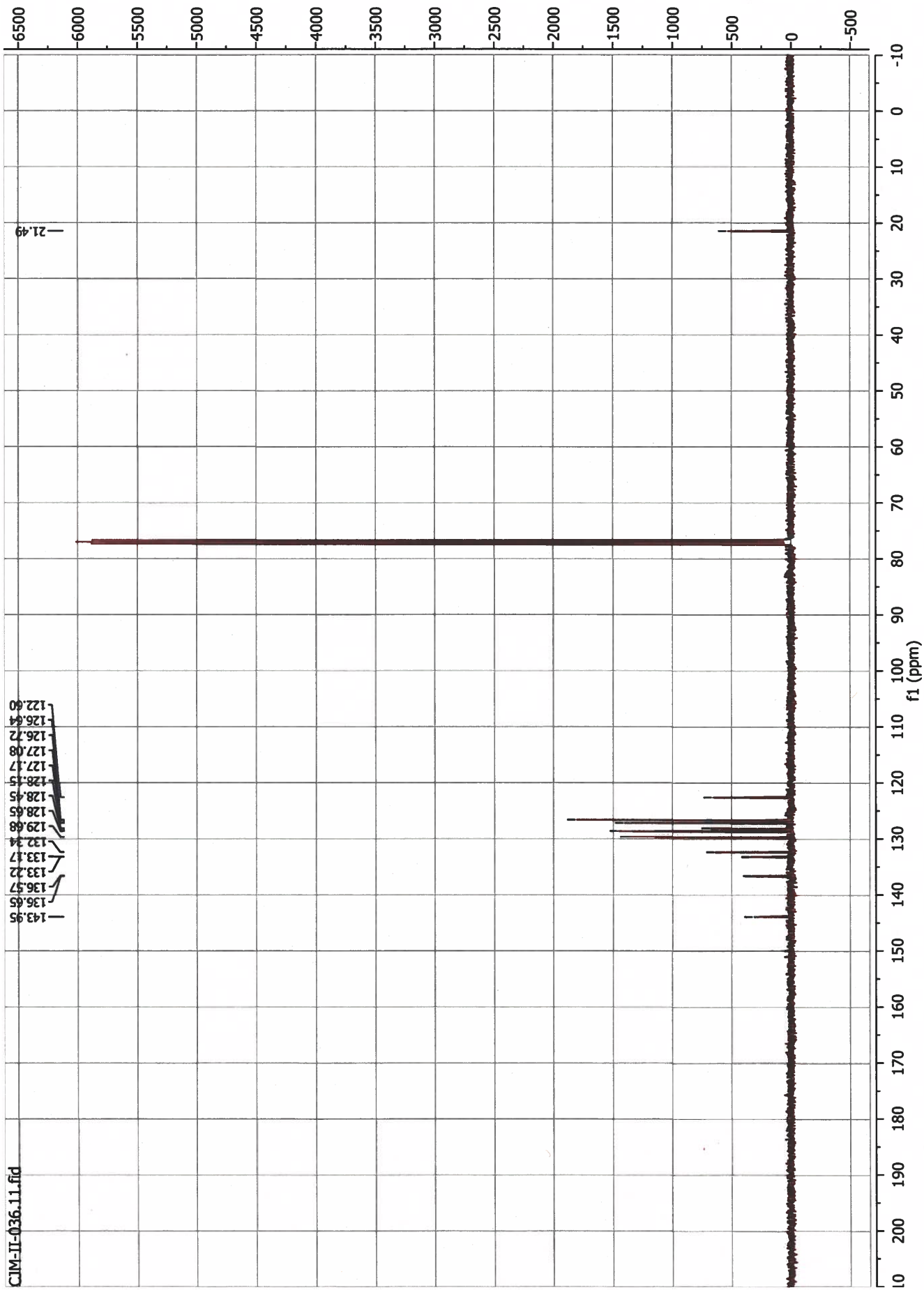

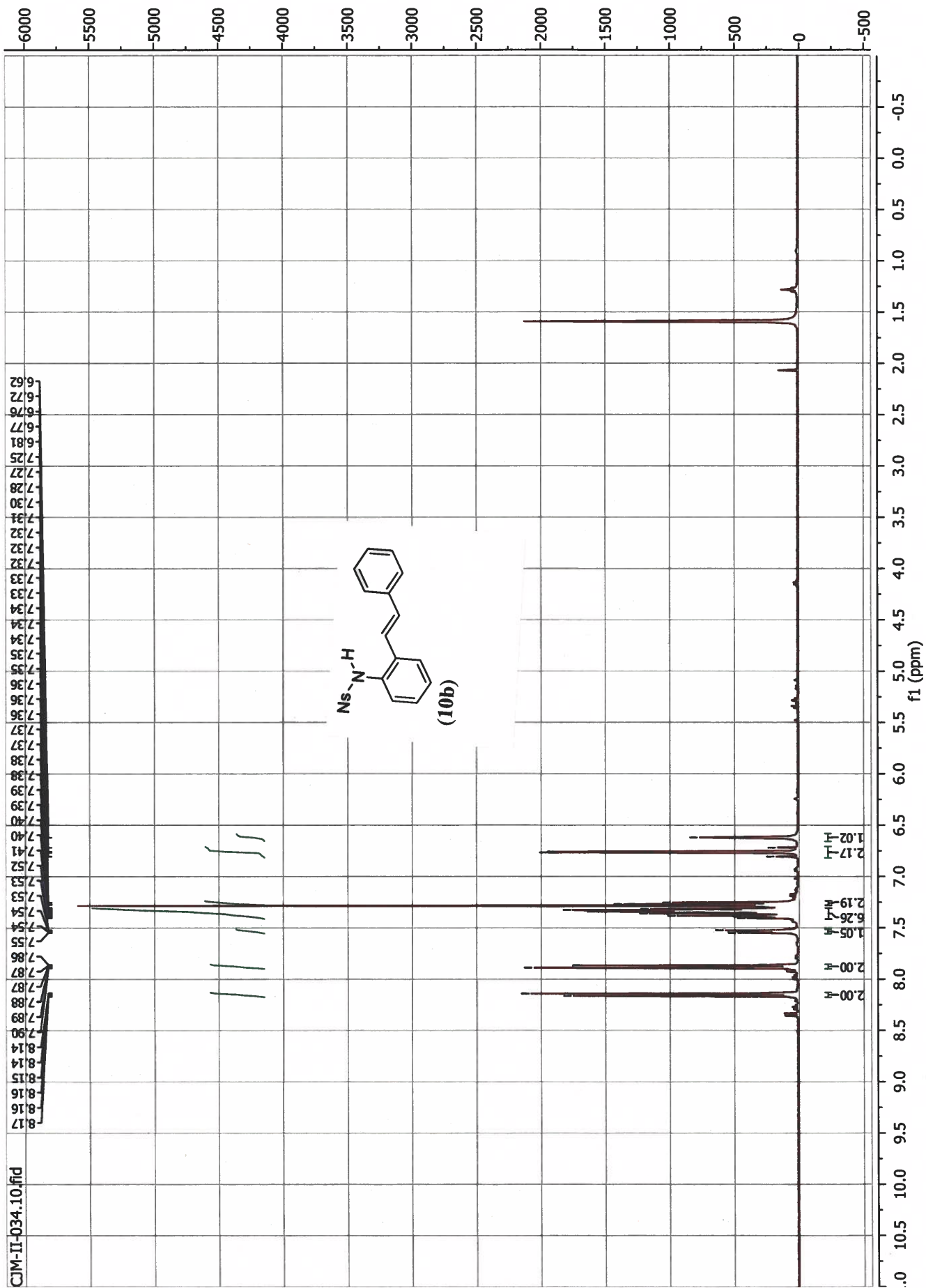

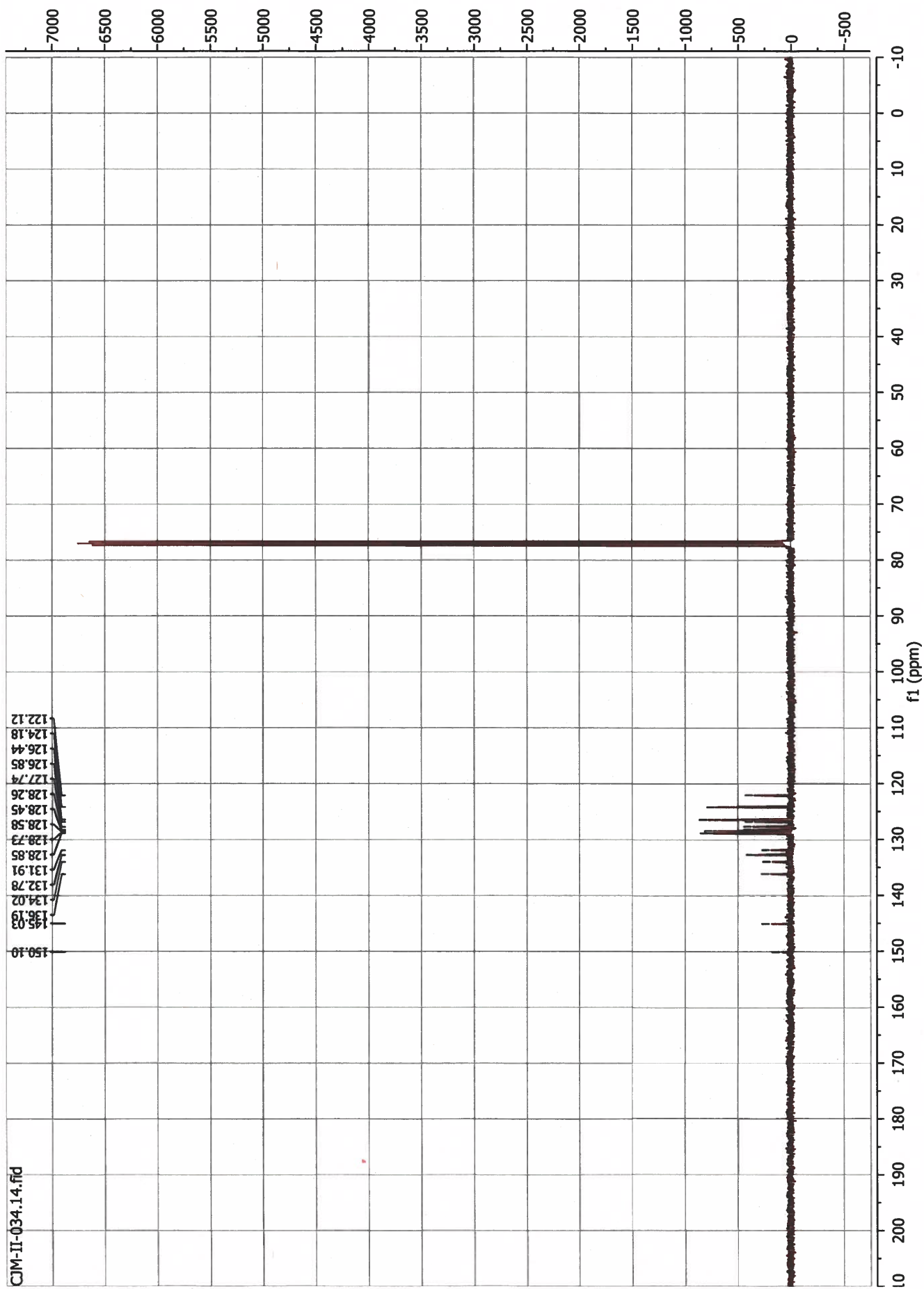

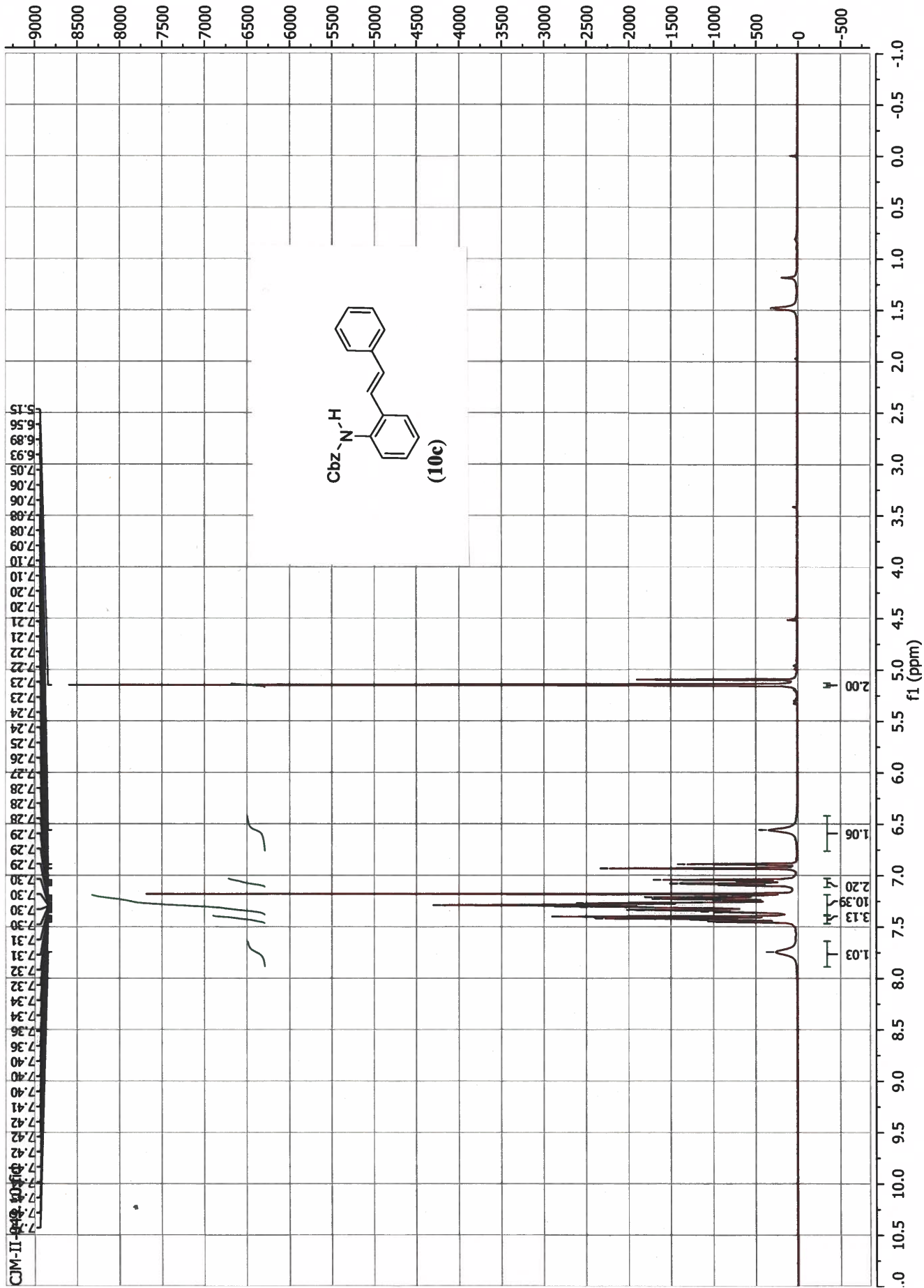

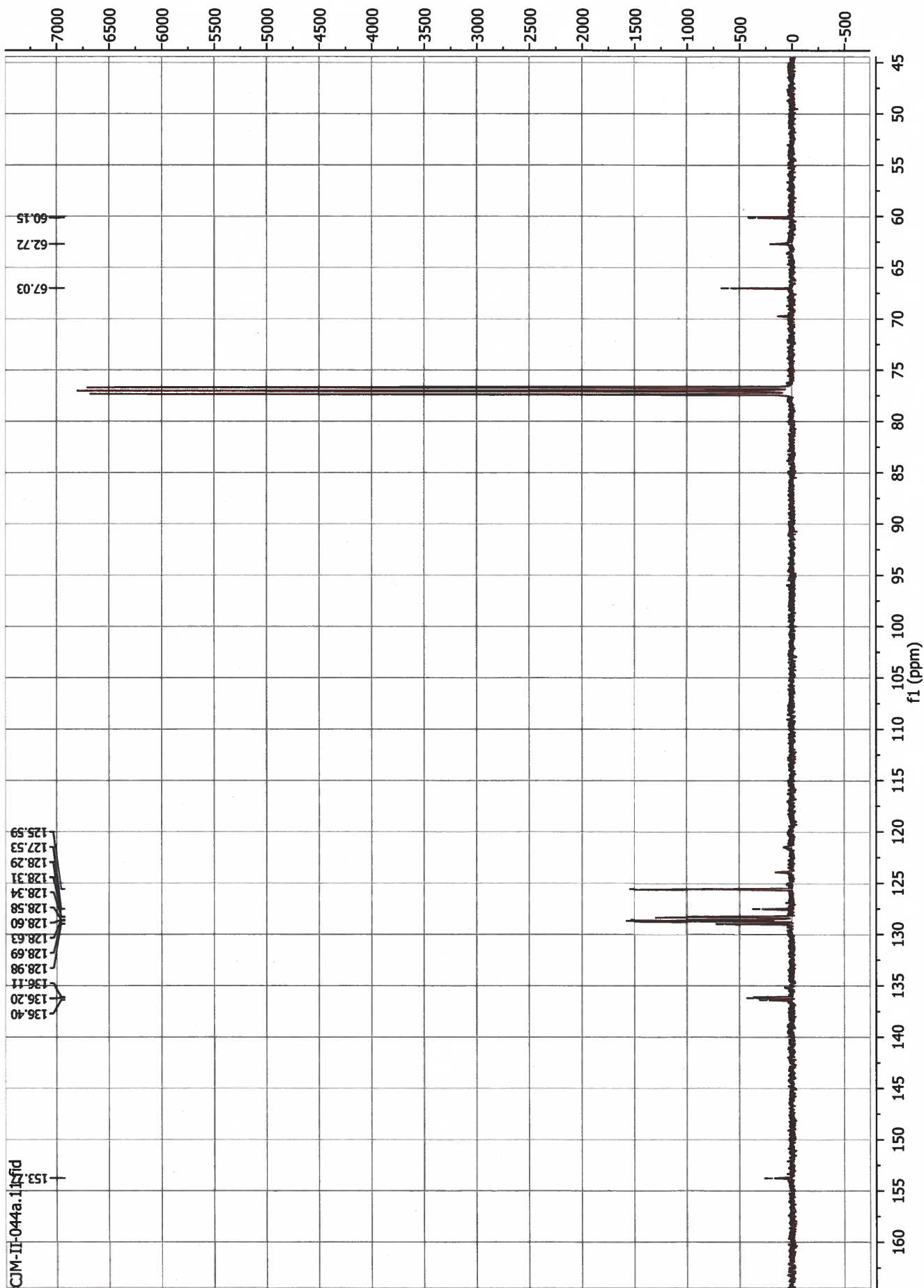

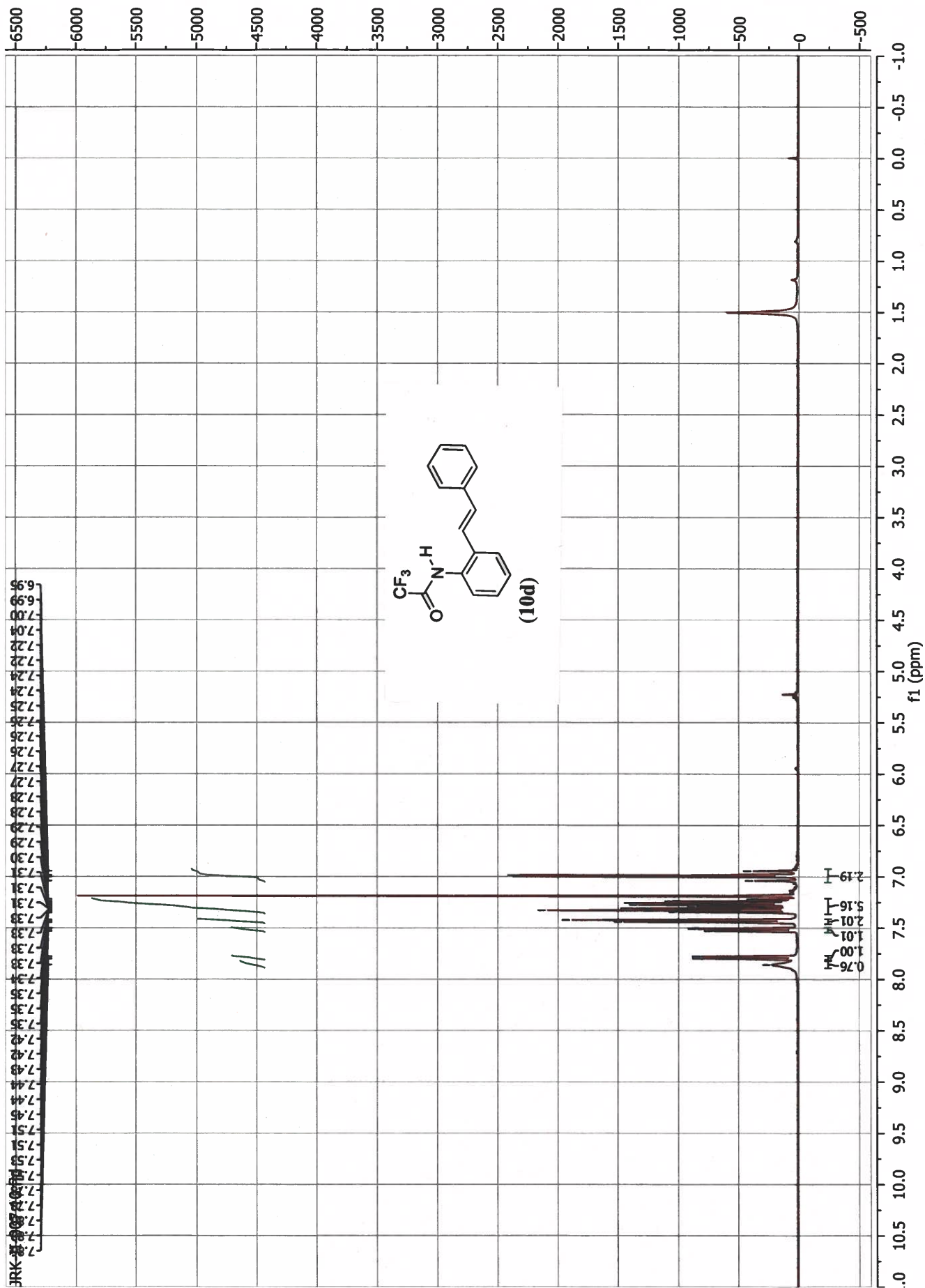

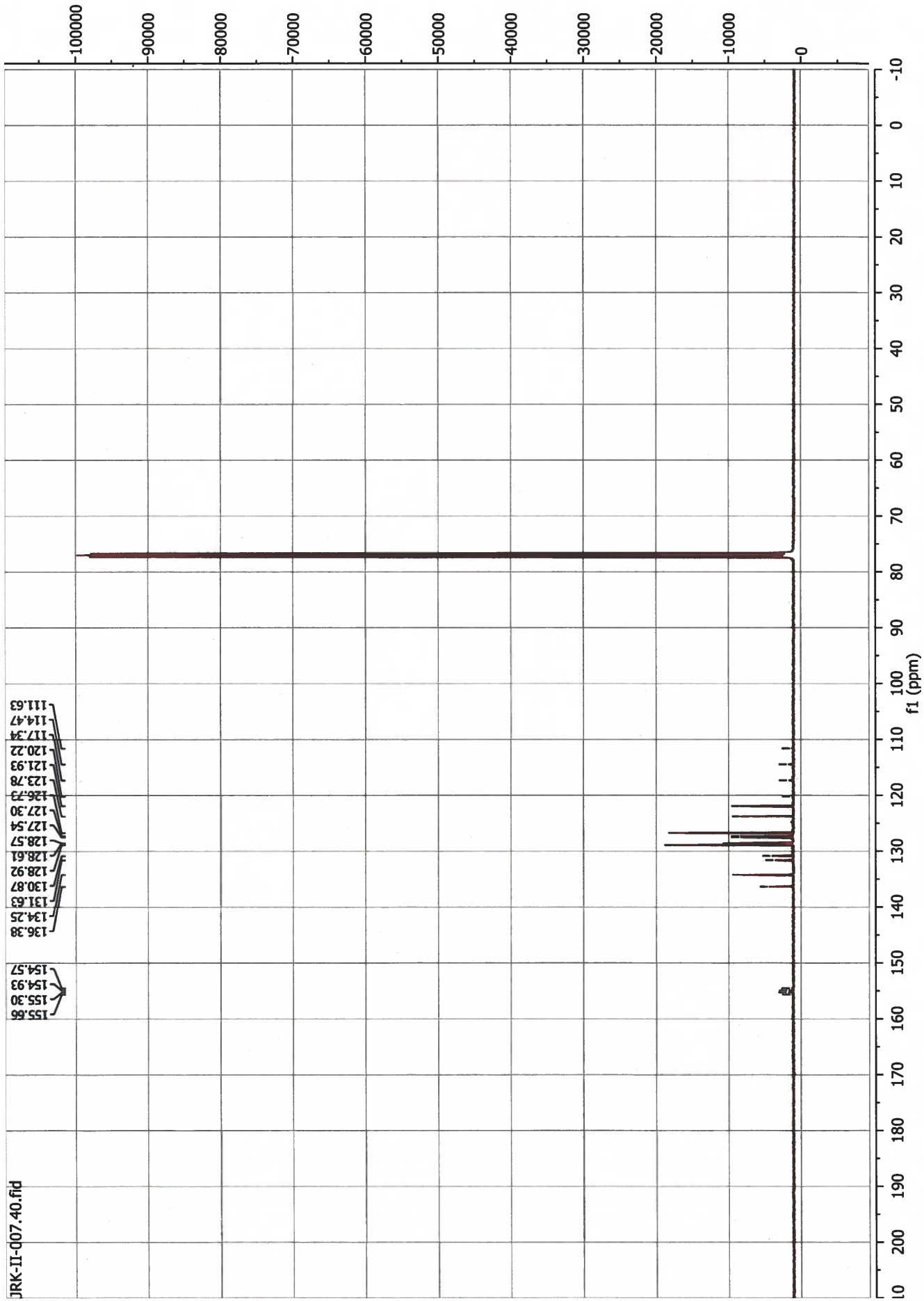

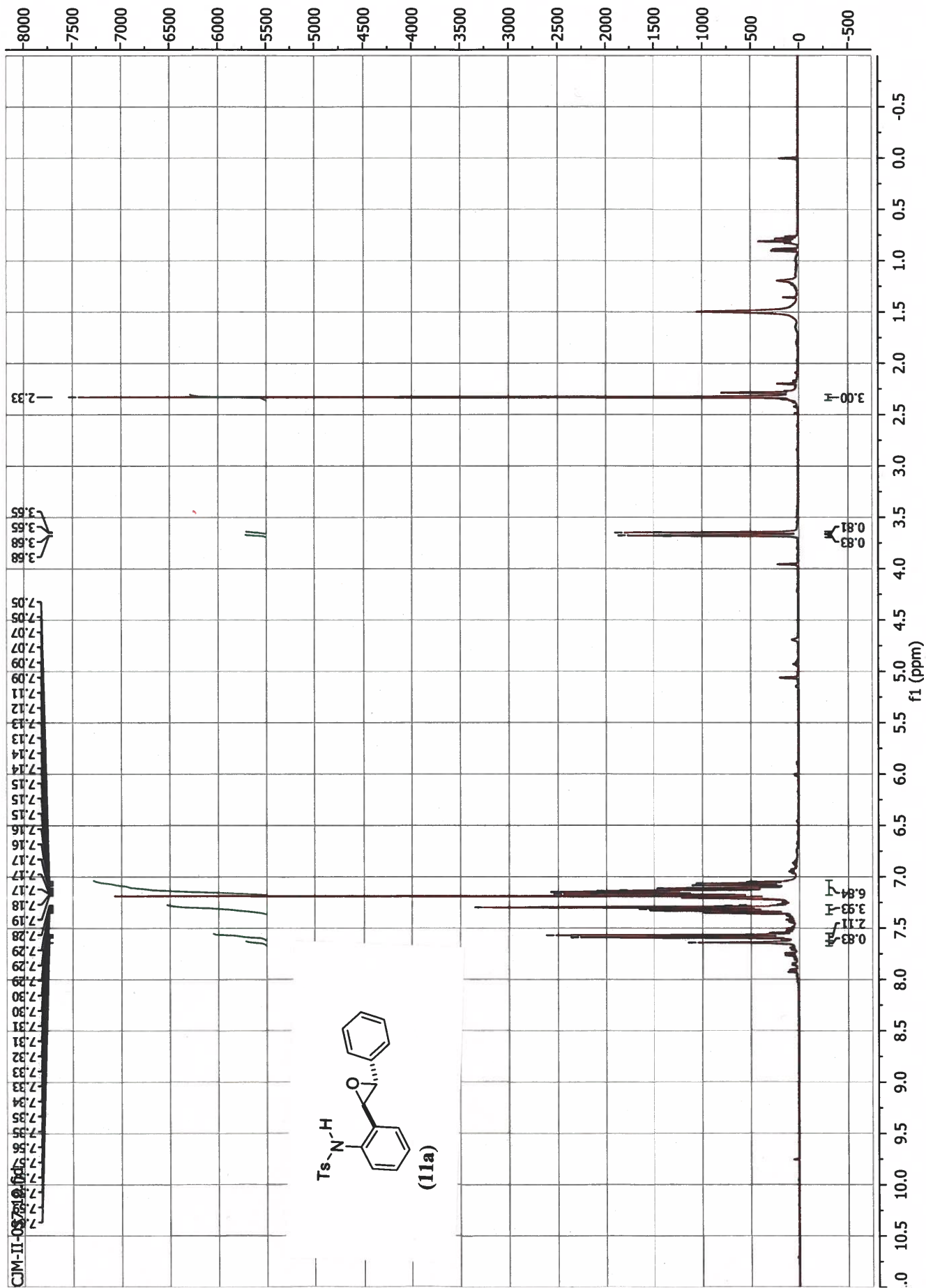

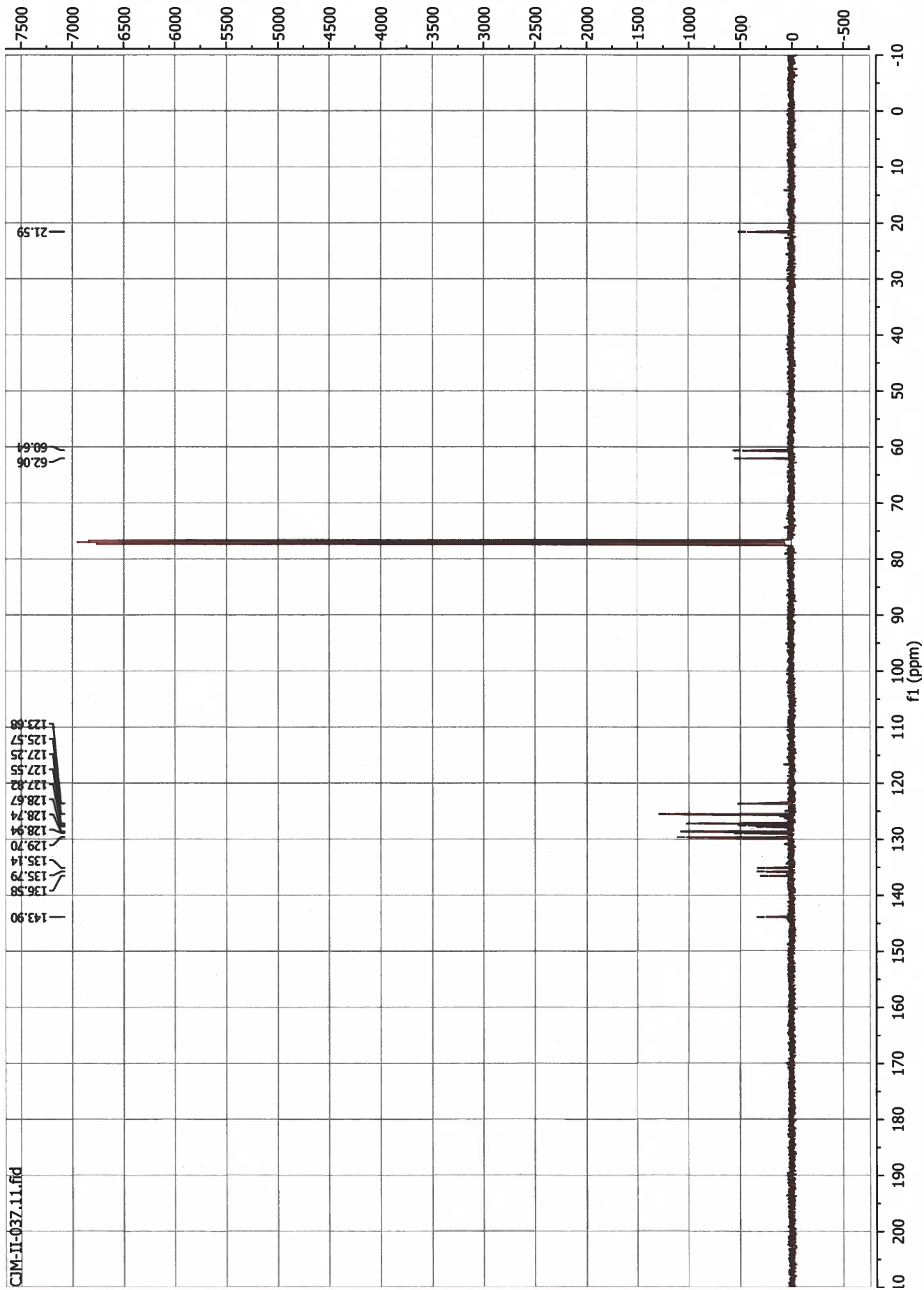

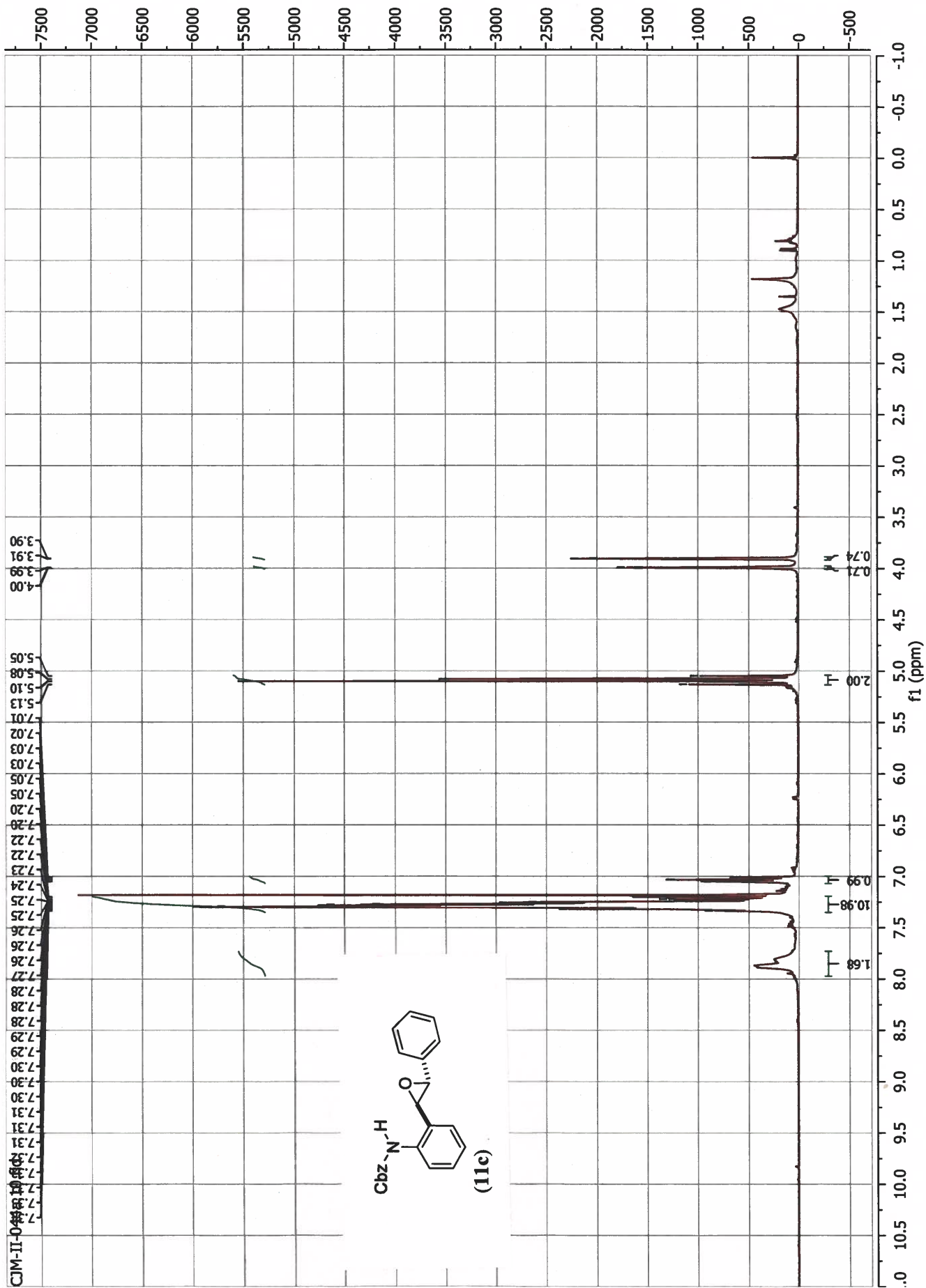

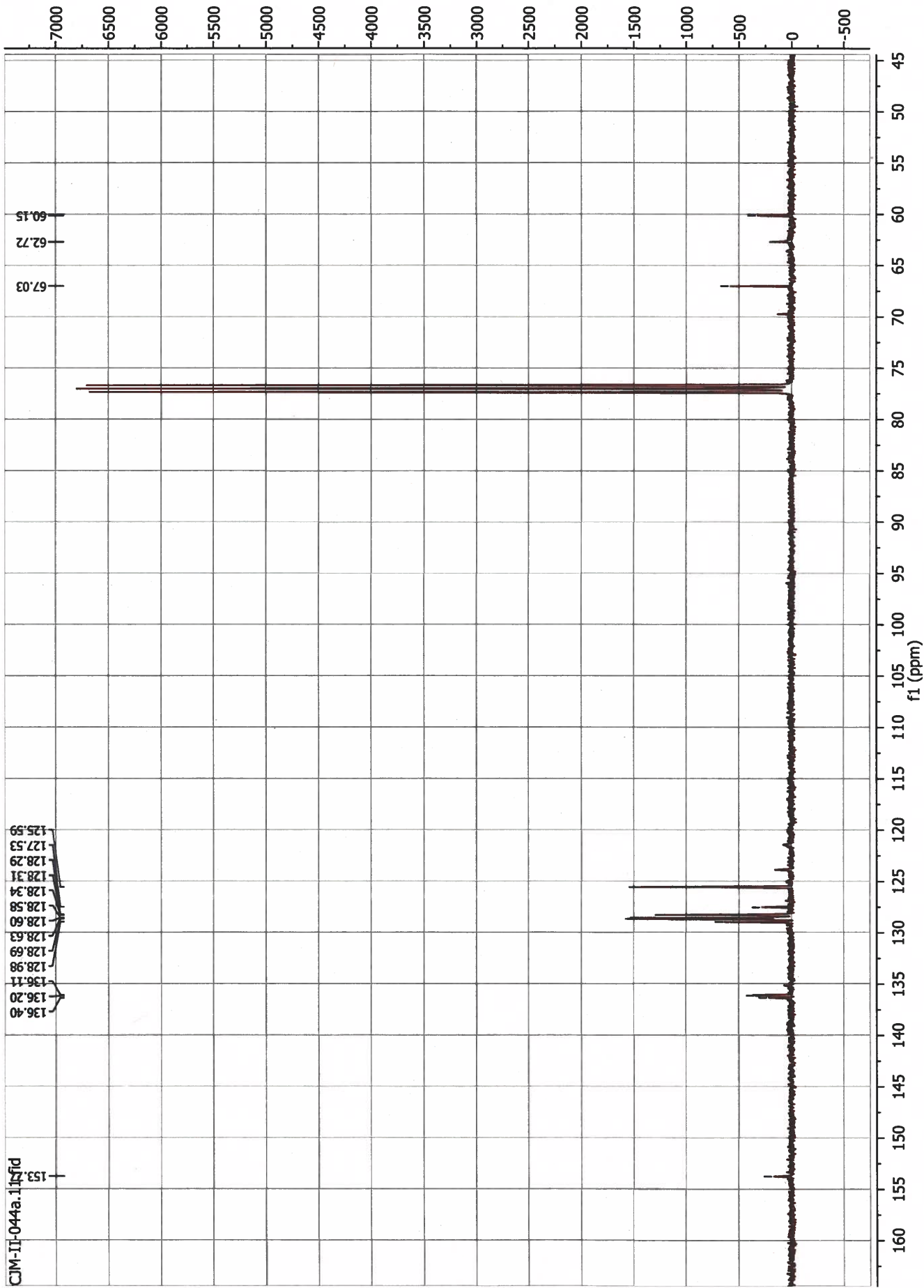

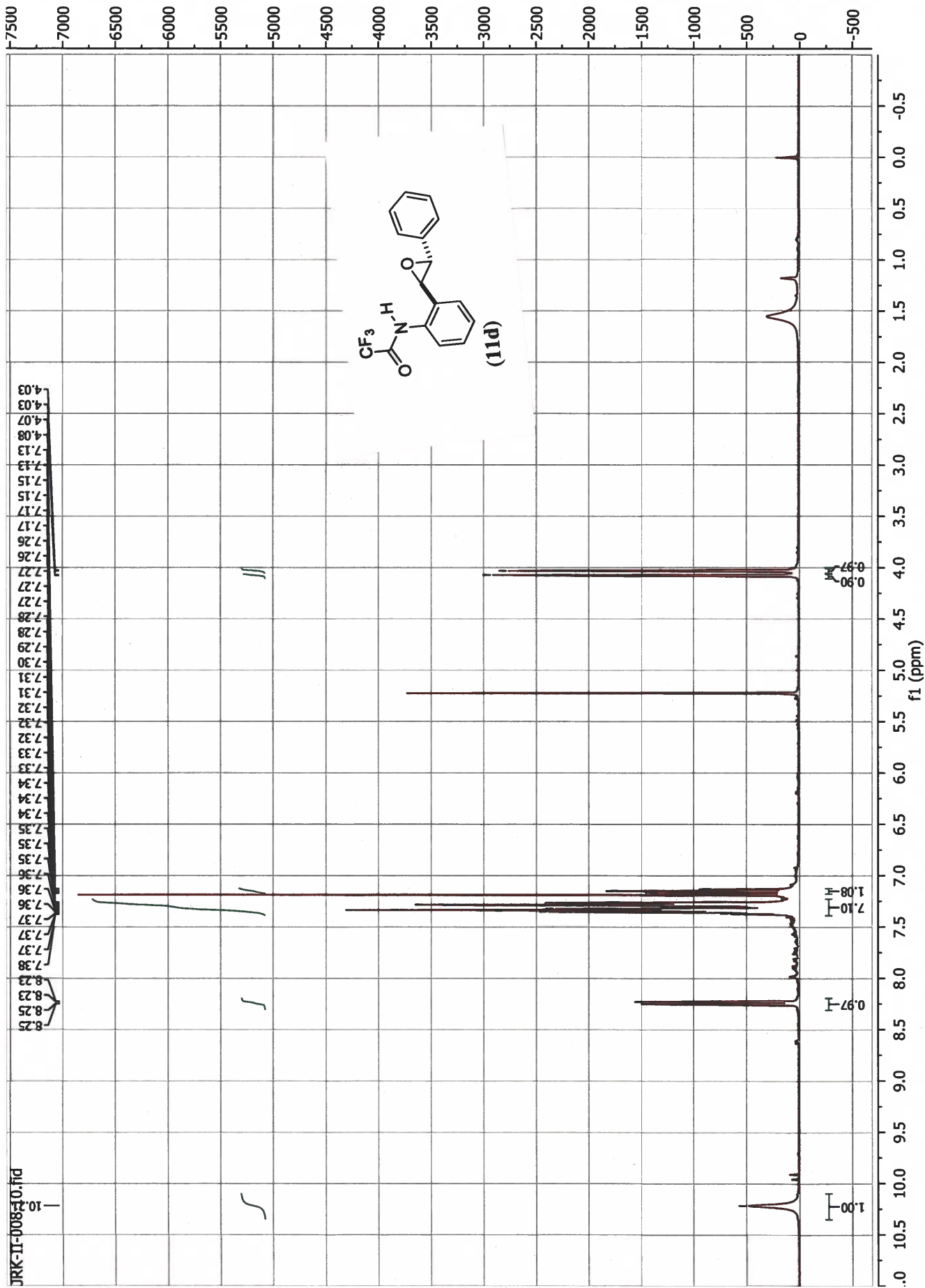

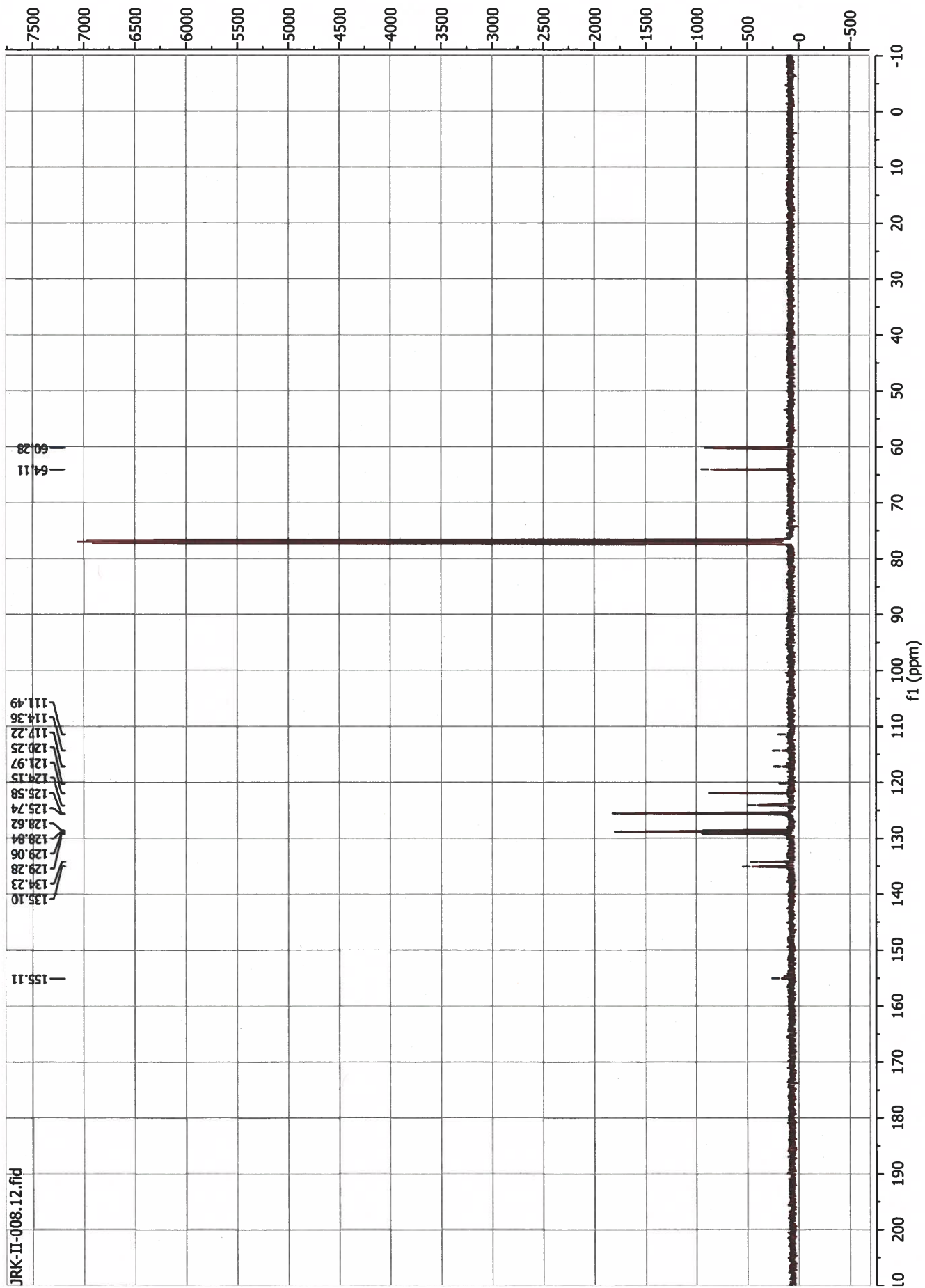

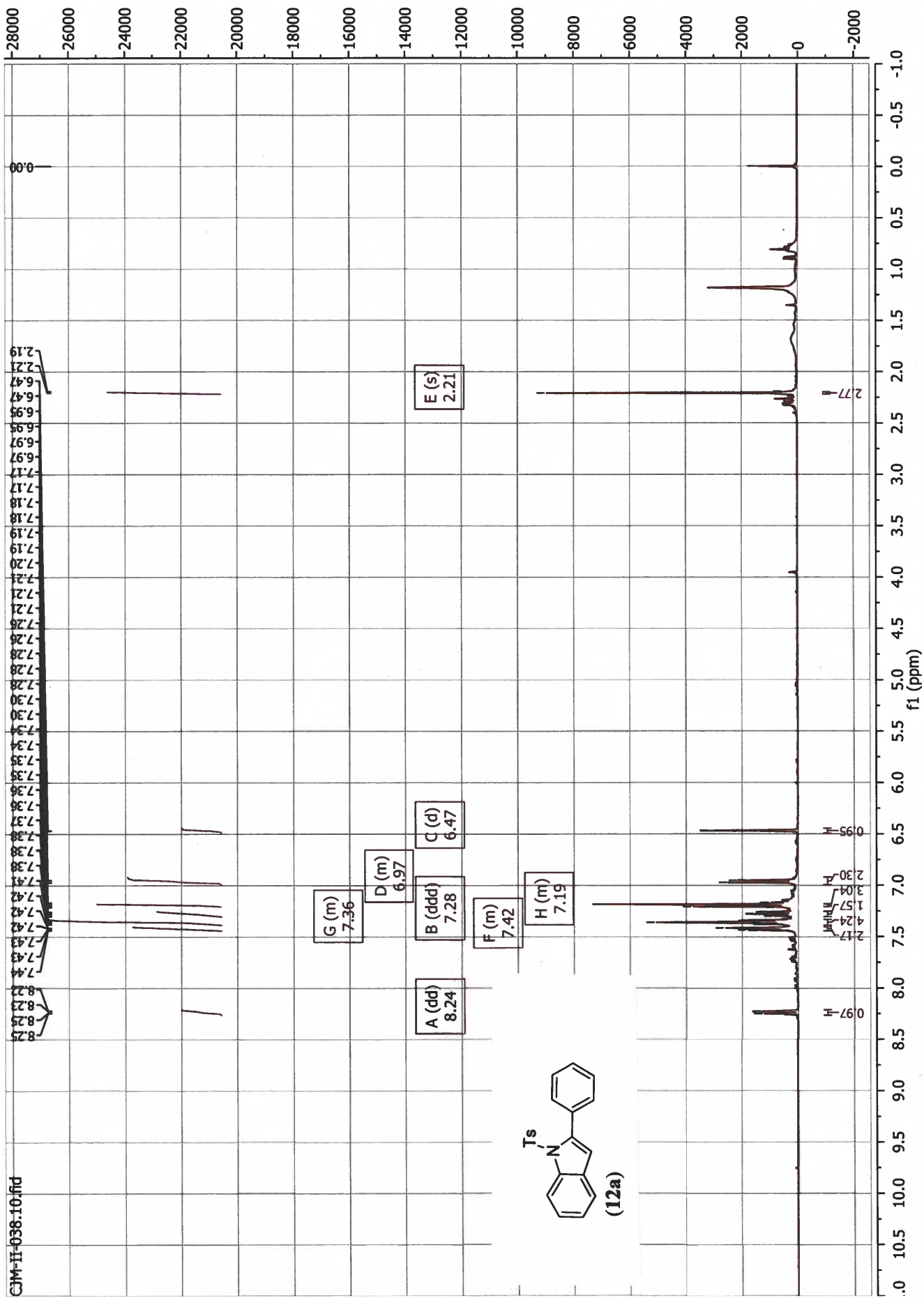

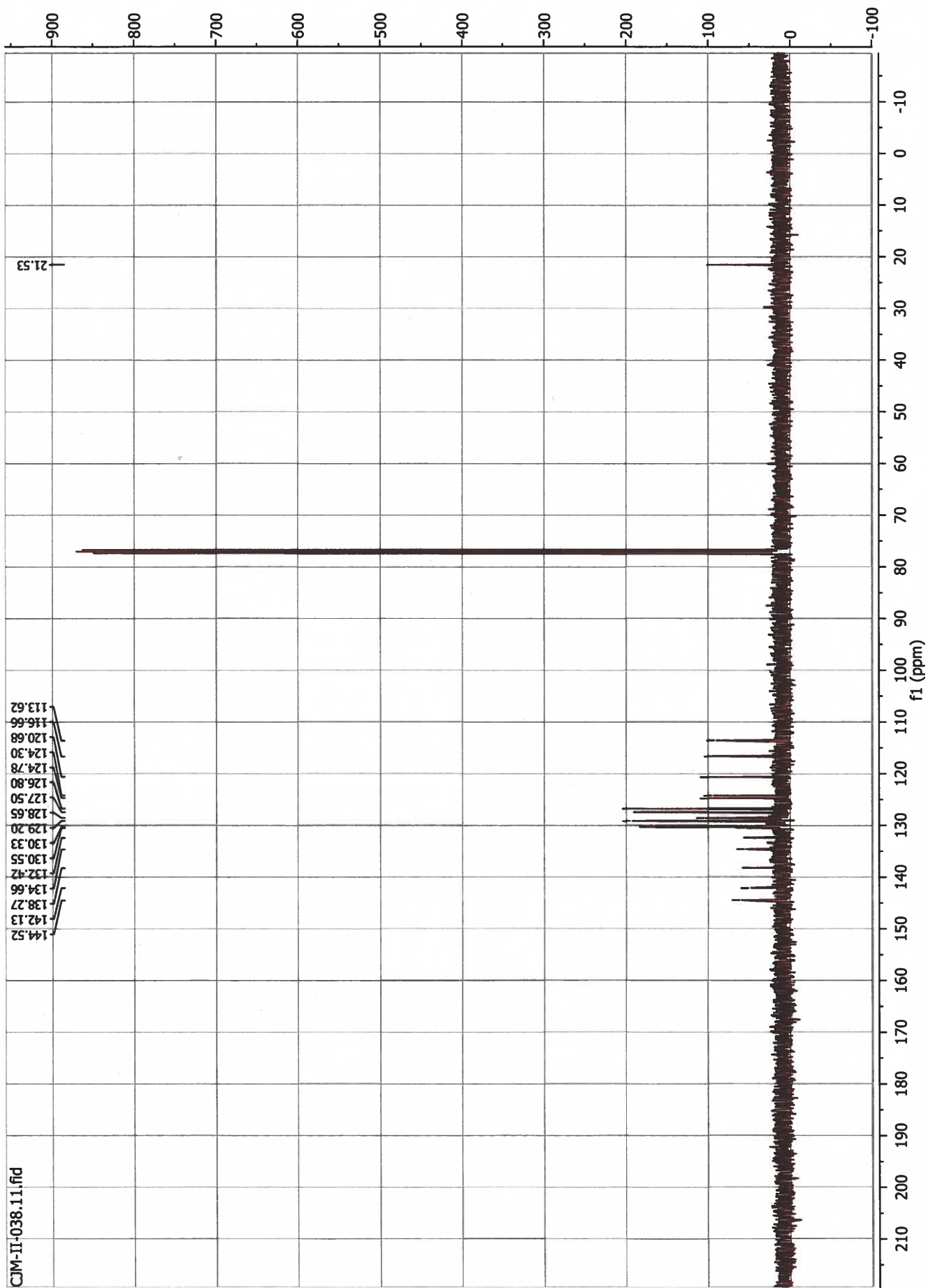

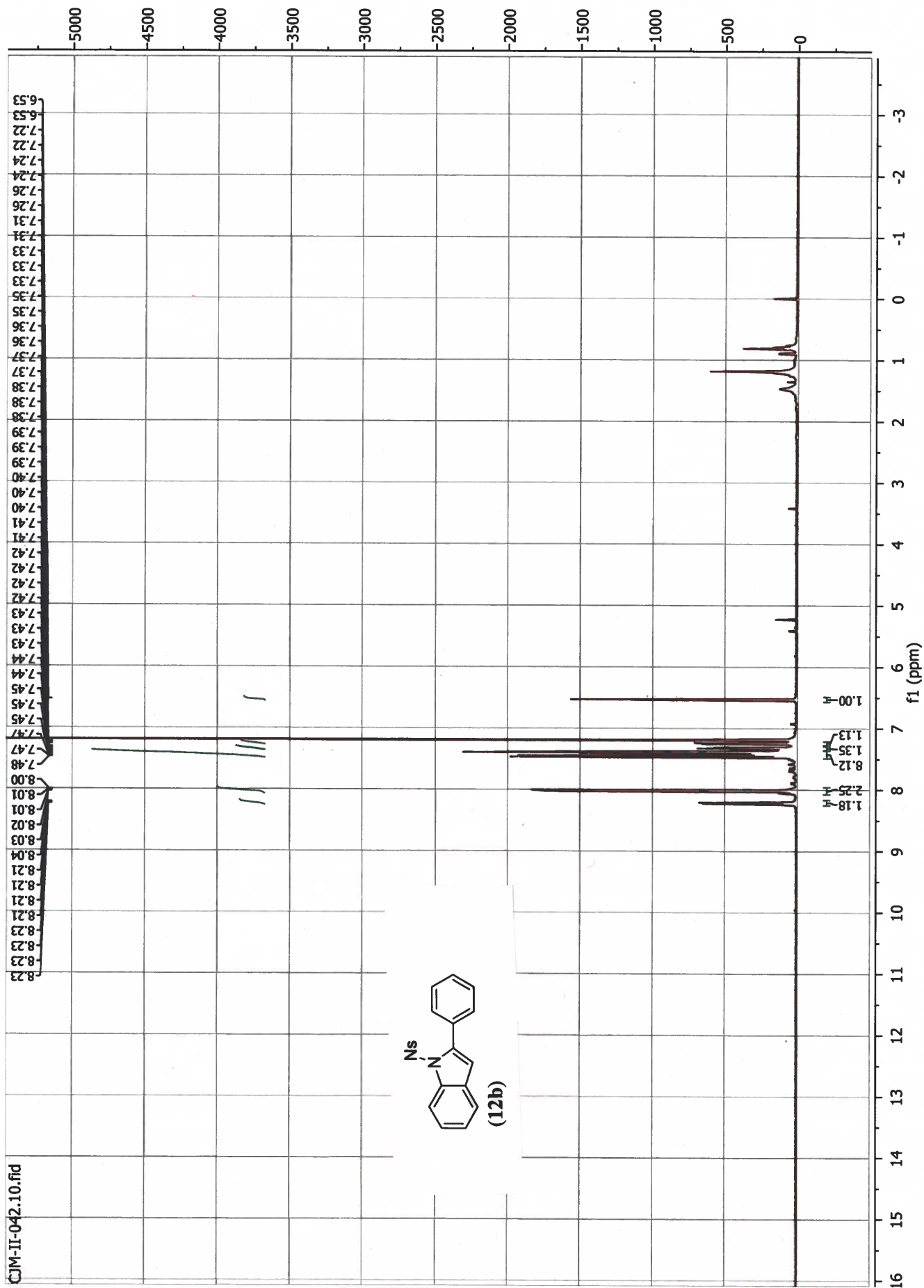

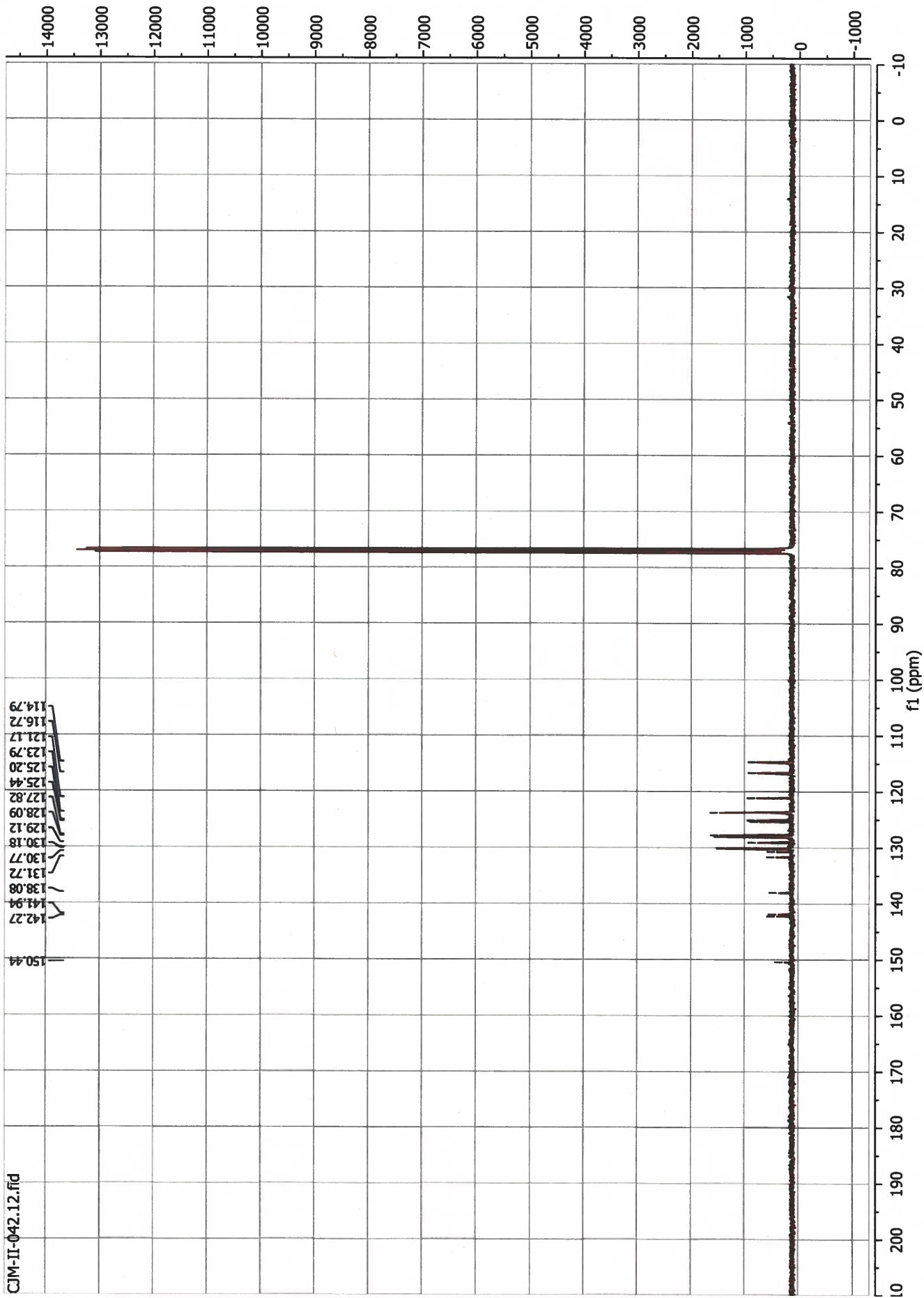

Supplement: Supplementary file 1 [file molecules-28-07968-s001.zip › molecules-2716472-supplementary.pdf]
